# Association between dietary caffeine, coffee, and tea consumption and depressive symptoms in adults: A systematic review and dose-response meta-analysis of observational studies

**DOI:** 10.3389/fnut.2023.1051444

**Published:** 2023-02-09

**Authors:** Kimia Torabynasab, Hossein Shahinfar, Nastaran Payandeh, Shima Jazayeri

**Affiliations:** ^1^Student Research Committee, Iran University of Medical Sciences, Tehran, Iran; ^2^Department of Nutrition, School of Public Health, Iran University of Medical Sciences, Tehran, Iran; ^3^Department of Community Nutrition, School of Nutritional Sciences and Dietetics, Tehran University of Medical Sciences (TUMS), Tehran, Iran

**Keywords:** caffeine, coffee, tea, depressive symptoms risk, observational studies, dose response meta-analysis

## Abstract

**Background:**

Recent studies have reported an association between dietary caffeine intake (coffee and tea) and the presence of depressive symptoms. However, the findings are not conclusive.

**Purpose:**

This study aimed to examine the correlation between the consumption of dietary caffeine (coffee and tea) and the presence of depressive symptoms in adults.

**Methods:**

PubMed and Scopus databases were searched until December 2021. Two investigators analyzed data from identified studies and rated the quality of the evidence using the GRADE approach. Using the random-effects models, we estimated the relative risks (RRs) and 95% confidence intervals (CIs). We also modeled the dose-response associations through a one-stage, weighted mixed-effects meta-analysis.

**Results:**

A total of 29 eligible studies included a total of 422,586 participants. On comparing the highest with the lowest category in cohort studies, we identified an inverse association between the intake of coffee and depressive symptoms (RR: 0.89, 95%CI: 0.82–0.95; I^2^ = 63.7%, GRADE = low). There was a 4% reduction in the risk of depression associated with an increase in coffee intake of 240 ml/day (RR: 0.96, 95%CI: 0.95, 0.98; I^2^ = 22.7%). By comparing the highest category with the lowest category in cohort studies, we discovered that caffeine intake was inversely associated with depressive symptoms (RR: 0.86, 95%CI: 0.79, 0.93; I^2^ = 0.0%, GRADE = moderate). Based on our data analysis, no correlation exists between tea consumption and depressive symptoms.

**Conclusion:**

According to our findings, coffee and dietary caffeine may have a protective effect against the development of depression. However, no evidence suggesting a link between tea consumption and reduced depressive symptoms has been found. Therefore, further longitudinal studies are needed to substantiate the causal relationship between coffee, tea, and caffeine and the risk of depression.

## 1. Introduction

As a leading cause of disability, depression has received considerable attention as a common mental disorder. The prevalence of depression has progressively increased in recent years, making it a public health concern throughout the world. According to a study by Our Word in Data, approximately 3.4% (with a margin of error of 2–6%) of the global population suffers from depression (1).

There are some strong associations between lifestyle factors and depression. Several studies suggest that an unhealthy lifestyle, including poor dietary patterns, alcohol consumption, and a sedentary lifestyle, substantially leads to the risk of depression (2–7). Other studies also demonstrated that obesity and depression have a bidirectional relationship, suggesting that depression can contribute to the development of obesity and that obesity can exacerbate the symptoms of depression (8). It was also indicated that inflammation and oxidation have a fundamental role in depression pathophysiology (9), and subsequently, attention was paid to anti-inflammatory compounds and antioxidant-rich foods as depression relievers. Studies conducted to examine the relationship between anti-inflammatory compounds and antioxidant-rich foods supported the mentioned hypothesis by exploring the effect of Mediterranean or plant-based dietary patterns on the risk of developing depression, as it resulted in a significant reduction (10–12). It is worth considering that a recent systematic review had also revealed evidence in support of the aforementioned studies showing that depression is negatively correlated with high adherence to diet recommendations, including avoiding processed foods, eating an anti-inflammatory diet, consuming magnesium and folic acid various fatty acids, and consuming fish (13).

Coffee and tea are among the most popular beverages in the world, and they are common sources of caffeine (14). It is suggested that caffeine is related to depression as it modulates dopaminergic transmission and facilitates serotonin release (15, 16). In addition to caffeine, there are some anti-inflammatory and antioxidant compounds in coffee, such as chlorogenic acid and catechins, which demonstrate potential beneficial effects against depression. In addition to caffeine, tea contains neuroprotective compounds such as L-theanine and polyphenols (17–19). A recent study also illustrated that during the COVID-19 pandemic, tea consumption increased (70 vs. 30%), effectively improving mood, focus, and performance and, as a result, relieving stress; this improvement is believed to be due to the presence of the compounds theanine and caffeine in tea (20).

Therefore, we performed a systematic review of observational studies to examine the correlation and the dose-response relationship between depression risk and the intake of coffee, tea, and caffeine.

## 2. Methods

### 2.1. Protocol and registration

We followed the Preferred Reporting Items for Systematic Reviews and Meta-Analyses (PRISMA-2020) guideline when we submitted this meta-analysis (21), and the protocol of the systematic review was registered at PROSPERO (registration number: CRD42022298824).

### 2.2. Literature search and selection

This investigation was carried out in accordance with the PRISMA reporting guidelines for systematic reviews and meta-analyses. The databases of PubMed and Scopus were systematically searched for relevant studies published in the English language up to December 2021. All the terms relevant to coffee, tea, caffeine, depression, and study design were applied to detect potentially eligible studies (Supplementary Table 1). We also checked the reference lists of the included studies and manually reviewed other studies to ensure that we did not miss any relevant studies.

### 2.3. Eligibility criteria

The inclusion and exclusion criteria were defined by adhering to the PICOS (population, intervention/exposure, comparator, outcome, and study design) framework. The criteria for inclusion were as follows: (1) cross-sectional, case-control, or prospectively designed studies conducted in the general population aged 18 years or older; (2) evaluating the association between coffee, tea, or caffeine intake as exposure and depression as the outcome; (3) assessing and reporting hazard ratios (HRs) or odds ratios (ORs) and the corresponding 95% confidence interval (CI) for ≥2 quantitative categories of dietary coffee, tea, and caffeine intake. For dose-response meta-analyses, it was also necessary to consider the number of cases and non-cases in each category of dietary exposures and ranges of intake. Studies reporting continuous estimation from the associations were also eligible. Review studies, interventional studies, and studies focusing on children, adolescents (under 18 years of age), and pregnant and lactating women were excluded.

### 2.4. Data extraction

Two independent investigators (KT and HSH) extracted the following data from the identified studies: the first author's last name, year of publication, country, and region where the study was conducted, the percentage of women who participated in the study, the sample size (total number of participants and number of cases), the study design, follow-up years, the age range of the study population at baseline, coffee, tea, and caffeine intake categories, the ORs with their 95% CI for each category of coffee, tea, and caffeine intake, exposure and outcome assessment tools, and covariates adjusted for in the multivariable analysis. We extracted the ORs that reflect the greatest degree of adjustment for potential confounders.

### 2.5. Quality assessment

Following the Robins-I tool framework, we evaluated the quality of the included (22). All studies were evaluated for confounding, participant selection, exposure assessment, misclassification during follow up, missing data, measurement of the outcome, and selective reporting of the results. The mentioned domains were classified as having a low, moderate, or serious risk of bias due to their characteristics. Supplementary Table 2 provides information about the domains of the Robins-I tool and how the judgment was made for each domain.

### 2.6. Statistical analysis

In the present meta-analysis, a random-effects model was performed to estimate the RR and its 95% CI as the effect size (23). For studies reporting the effect size as the odds ratio or hazard ratio, we considered them to be equal to HR (24). In the first step, a meta-analysis was conducted to compare the risk ratios of caffeine, coffee, and tea consumption in primary studies based on high vs. low intake categories. Whenever there are sex-specific effect sizes, we used a fixed-effect model to combine the estimates and then applied the combined effect size to the analysis. Cochran's Q (25) and I^2^ statistics (26) were used to test for heterogeneity.

To identify potential sources of heterogeneity, subgroup analyses were conducted based on geography, follow-up duration, number of participants, and adjustments for main confounders such as energy intake, body mass index (BMI), smoking, drinking alcohol, and exercising. With at least ten primary studies available, we used Egger's test to determine the presence of publication bias (27) in addition to visually inspecting funnel plots (25). Additionally, we conducted a sensitivity analysis to assess the impact of individual studies on the overall estimate by excluding them one by one.

Based on the method presented by Greenland and colleagues, we also conducted a random-effect dose-response meta-analysis for studies whose data were sufficient for dose-response meta-analyses of depression for a specific amount of increase in coffee, caffeine, and tea consumption (28, 29). It was required to calculate the median point in each category, as well as the distribution of cases and person-years based on dietary caffeine, coffee, and tea. Our final approach was to perform a one-stage linear mixed-effects meta-analysis to clarify dose-response relationships (30). Based on Harrell's recommended percentiles of 10, 50, and 90%, we used restricted cubic splines with three knots to model the exposures (31). We combined the study-specific estimates by using a one-stage linear mixed effects meta-analysis to take into account correlations within each category of relative risks (30). As a result of this method, the study-specific slope lines were estimated in a single step and combined to obtain an overall average slope (28, 32). Due to the limited number of studies (*n* ≤ 2) included in the analysis, we used the best-fitting second-order fractional polynomial to model curvilinear associations (30). Statistical analyses were conducted using STATA version 16.0. A *P*-value of less than 0.05 was considered significant.

### 2.7. Grading the evidence

The quality of the evidence was assessed by two independent authors (HSH and KT) using the GRADE approach (33). This tool classifies evidence as strong, moderate, low, or very low quality. The evidence used in this study was classified as low quality, as it was based on observational studies such as prospective cohort studies and cross-sectional studies. Depending on pre-specified criteria, they can be downgraded or upgraded. Evidence that has been downgraded includes limitations of the study, inconsistency, indirectness, imprecision, and publication bias, whereas evidence that has been upgraded includes a significant degree of association, a dose-response gradient, and attenuation caused by plausible confounding. Disagreements were resolved with a consensus.

## 3. Results

A total of 1,348 studies were identified at baseline from databases (PubMed 295 and Scopus 1,053), and five articles were found through manual search. We removed 228 duplicate studies. Of the 1,125 remaining studies, 1,063 were excluded on the basis of title and abstract screening. Sixty-two articles were screened by reading their full text. Of those, 33 were excluded as 11 were review articles and 22 articles reported irrelevant outcomes. Ultimately, our systematic review ultimately included 29 studies that met the inclusion criteria established for this study. The process through which studies were selected and identified is summarized in Supplementary Figure 1.

### 3.1. Study characteristics

The study included a total of 422,586 participants, ranging in age from 18 to 97 years. Eligible studies were published between 2005 and 2021. Table 1 shows detailed information extracted from articles. Among them, five studies considered coffee consumption as the main variable, seven studies considered tea (39, 41, 42, 44, 49, 58, 61) or coffee (40, 45, 51, 53, 62) as the main variable, seven considered caffeine (35, 38, 54, 55, 63), and 10 studies considered coffee, tea, or caffeine altogether (34, 36, 37, 43, 46, 48–50, 52, 56, 57). In addition, twenty studies had a cross-sectional design (34–36, 38, 42, 43, 45, 48–52, 54–57, 61–64), seven studies were prospective cohorts (37, 39, 40, 44, 46, 53, 58), and two studies evaluated both cross-sectional and prospective data (44, 47). Depression was identified by validated depression questionnaires such as the Beck Depression Inventory (BDI) (34), the Geriatric Depression Scale (GDS) (36, 41, 50), the Center for Epidemiological Studies Depression Scale (CES-D) (39, 42–44, 56, 61, 64), the Patient Health Questionnaire (PHQ-9) (49, 51, 54, 55), the Hospital Anxiety and Depression Scale (HADS) (38, 62), the Depression Anxiety Stress Scale (DASS-21) (48), and the Minimum Data Set-based Depression Rating Scale (MDS-DRS) (63). In 21 studies, depression was assessed using standardized scales, while in other studies, varied methods were used, such as physician diagnosis (40, 46, 53), self-report of depression (40, 45, 46, 57), the International Classification of Diseases (ICD) criteria (37), the Diagnostic and Statistical Manual of Mental Disorders (DSM) criteria (35), the MINI (Mini-International Neuropsychiatry Interview) (47), and antidepressant use (40, 53). Coffee, tea, and caffeine intake were assessed with validated questionnaires such as the Food Frequency Questionnaire (FFQ) (40, 45, 46, 53, 57, 62), the 4-day food record (37), 24-hr recall (54, 55, 64), the Brief Dietary History Questionnaire (BDHQ) (43, 56), an interview or by asking participants (34–36, 38, 39, 42, 44, 47–52, 61, 63), and observation as well (58). Studies have been carried out in Finland (34, 37), Virginia (35), Japan (43, 56), the United Kingdom (38, 57), China (39, 42, 49, 50, 61), the United States (40, 46, 54, 55, 64), Japan (36, 57, 62), Taiwan (44), Korea, France (47), Tripoli (48), Atlantic Canada (51), Korea (45, 52), Navara (53), the Netherlands (57, 63), Germany (57), Spain, Singapore (41, 58), and Palestine (60). By gender, three studies included only women (39, 40, 56), and one study included only men (37), while 25 other studies included both genders (34–36, 38, 41–46, 48–52, 54, 55, 57, 58, 61–64).

**Table 1:** Characteristics of included studies.

**References**	**Country**	**Female, %**	**Age range (year)**	**Design**	**Sample size**	**Cases**	**Exposure**	**Effect size (95%CI)**	**Comparison**	**ExposureAssessment**	**Exposure (range of intake)**	**OutcomeAssessment**	**Follow-up (year)**	**Adjustments**
Hintikka et al. (34)	Finland	55.7	25–64	Cross-sectional	2,011	210	Coffee Tea	Tea: 0.47 (0.27–0.83) Coffee: 0.90 (0.54–1. 50)	Daily (≥5 cups/day) vs. Not daily	Questionnaire	0–≥5 cups/day	21-item BDI	1998–2005	Age, sex, current daily smoking, alcohol consumption patterns, marital status, employment status, length of basic education, having vocational training, economic hardship and poor subjective health, frequency of eating lake fish, sea fish, fresh vegetables, boiled vegetables, and fruits and use of multivitamin pills and fish oil capsules
Kendler et al. (35)	Virginia	51.8	37.9	Cross-sectional	3706	NA	Caffeine	1.79 (1. 47–2.17)	At least several days per week vs. ≥625 mg of caffeine per day	Interview	0–>650 mg/day	DSM-III-R criteria	1995–1997	Age, gender
Niu et al. (36)	Japan	57.3 607 women	>70	Cross-sectional	1,058	361	Coffee Green tea Black or oolong tea:	Coffee: 0.82 (0.53– 1.27 Green tea: 0.56 (0.39– 0.81) Black or oolong tea: <1 cup/d: 0.82 (0.56– 1.20) ≥1 cups/d: 0.71 (0.49, 1.02)	NA	Questionnaire	Coffee: Almost never–≥1 cups/d Tea: ≤1–≥4 cups/d	30-item GDS	2002–2009	Age, sex, BMI, hypertension, diabetes, history of cardiovascular diseases, cancer, or arthritis, high C-reactive protein, smoking and drinking habits, physical activity, cognitive status, impaired instrumental activities of daily living, body pain, education, living alone, marital status, serum albumin concentration, total energy intake, intakes per 2,000 kcal of energy intake as protein and folate, tea consumption (for coffee analysis), coffee consumption (for tea analysis), perceived social support, visiting friends
Ruusunen et al. (37)	Finland	0	42–60	Prospective cohort	2,232	49	Coffee Tea Caffeine	Coffee 0.25(0.07- 0.91) Tea 1.40 (0/78- 2.51) Caffein 0.85 (0.34–2.15)	Coffee: >813 ml/day vs. never Caffeine: > 781 mg/d	4d record	Coffee: none->813 ml/d Tea: yes/no Caffeine: >425 mg/d-781 mg/d	Diagnosed by a physician by ICD criteria ICD-9	17/5 years	Age, examination years, socio-economic status, smoking, alcohol consumption, maximal oxygen uptake, BMI, and daily intakes of folate and PUFA
Smith (38)	United Kingdom	57	49.6	Cross-sectional	3,223	NA	Caffeine	0.12 (0.1- 0.2)	>250 mg/day	Questionnaire	<140–>260 Mg/d	HADS	NA	NA
Chen et al. (39)	China	100	53.7	Prospective cohort	1,399	363	Tea	0.39 (0.19 to 0.84)	>100 g dried tea leaves/ mo. vs. never	Interview	0 to >100 g dried tea leaves	20-item CES-D	2002–2006	Age at diagnosis, education, income, marital status, exercise, comorbidity, menopausal symptoms, relapse/metastasis, radiotherapy, and quality of life (SF-36 mental health index scale score)
Lucas et al. (40)	USA	100	63	Prospective cohort	50,739	2,067	Coffee	0.82 (0.68–0.98)	≥4 cups/day vs. ≤1 cups/week	FFQ	0–≥4 cups/day	defined as self-reported physician-diagnosed depression and antidepressant use	1996–2006	Age, interval, total energy intake, menopausal hormones use, smoking, BMI, physical activities, marital status, social or community group involvement, self-reported history of diagnosis of diabetes, cancer, myocardial infarction or angina, high blood pressure, MHI score, a minimum latency of exposure of 8 years
Feng et al. (41)	Singapore	NA	55–93	Prospective cohort	1,615	73	Tea	0.30 (0.11–0.85)	≥6 cups/day vs. never	Interview	0–≥6 cups/ day	GDS-15	2005–2007	Age, education, housing type, marital status, physical exercise, social and productive activities summed score, MMSE total score, GDS total score
Feng et al. (42)	China	59.3	68.6	Cross-sectional	1,368	285	Tea	0.58 (0.42–0.80)	No or irregular consumption per month–daily consumption	Asking participants	No or irregular consumption per month–daily consumption	15-item CES-D	June 2010 to July 2011	Age, education, housing type, marital status, physical exercise, social and productive activities summed score, MMSE total score, GDS total score
Pham et al. (43)	Japan	40% 218 women	20–68	Cross-sectional	537	157	Coffee Green tea Caffeine	Coffee: 0.61(0.38- 0.98) Green tea: 0.54 (0.29- 1) Caffeine: 0.57 (0.30–1.05)	Coffee: ≥2 cups/day vs. <1 cup/day Caffeine: ≤100 mg/d–>291 Green tea: ≥4 cups/day vs. ≤1 cup/day	BDHQ	Coffee: <1–≥2 cups/day Caffeine: ≤100 mg/day- >291 mg/day Green tea: ≤1–≥4 cups/ day	20-item CES-D	2009–2013	Age, sex, workplace, cancer, CVD, diabetes or chronic hepatitis, marital & living status, overtime work, BMI, job position, smoking, physical activity, alcohol drinking, n-3 PUFA · red meat · vegetable · fruit · coffee · green tea consumption, serum C-reactive protein concentration, serum folate concentration
Tsai et al. (44)	Taiwan	46.8	≥53-year-old	Prospective cohort Cross-sectional	Longitudinal: 2,145 Cross sectional: 4,122	Longitudinal: 31/8%= 682 Cross sectional: 36.8%= 1,516	Tea	Longitudinal: 0.83 (0.65–1.08) Cross-sectional: 0.63 (0.50–0.79)	≥3 times/ week vs. ≤2 times/week	Interview	≤2 to ≥3 times/week	10-item CES-D	1999–2007	Age, sex, level of education, psychological stress, diabetes, heart disease, IADL status, family support, audio acuity
Park and Moon (45)	Korea	59.6% 6,069 Women	20–97	Cross-sectional	10,177	425	Coffee	0.58 (0.44–0.76)	≥3 cups/day vs. ≤ 0.14 cups/day	FFQ	0.14–3 cups/d	Self-reported depression	2010–2011	Diseases and stroke, perceived stress level, coffee · green tea · soft drink · vegetable · fruit · blue-backed fish · bean · red meat consumption
Omagari et al. (36)	Japan	13.3	41–82	Cross- sectional	89	15	Coffee	0.082 (0.009–0.711)	Coffee: 0–2 vs. ≥3 cups/d	FFQ	0–≥3 cups/day	HADS	April to September 2013	Sex, lipids, and n-6 PUFAs, the lipid and carbohydrate energy ratios
Guo et al. (46)	USA	51.5	50–71	Prospective cohort (nested case-control)	252,612	11,311	Coffee Tea	Tea: M: 1.21 (0.95–1.53) F:1.01 (0.92–1.32) Coffee M:0.90 (0.80–1.01) F:0.93 (0.84–1.04)	None vs. ≥4 cups per day	FFQ	0–≥4 Cups/day	self-reported physician-diagnosed depression	1995–2006	Age, sex, race, education, marital status, smoking, alcoholic beverage intake, physical activity, BMI, energy intake
Ritchie et al. (47)	France	Cross-sectional: 61.3 longitudinal: 56.3	≥65	Cross-sectional longitudinal	Cross-sectional: 8,125 longitudinal: 5,785	Cross-sectional: 1,973 longitudinal: 1,076	Caffeine	Cross-sectional: M: 0.94 (0.76- 1.18) F:0.92 (0.80- 1.06) Longitudinal: M: 0.85 (0.66- 1.08) F: 0.86 (0.74- 1.01)	No comparison	Interview	≥3 Cups/day (≥3 units of caffeine, each unit = 100 mg 1 cup of coffee = 100 mg 1 cup of tea = 50 mg)	MINI	NA	Age and center, education, cardiovascular pathologies, hypertension, BMI, HDL cholesterol, triglycerides, mobility, baseline depressive symptoms
Taher et al. (48)	Tripoli	68.5	38.7 ± 8.5	Cross-sectional	200	89	Tea or coffee	2.48 (1.36–4.54)	Yes/no	Questionnaire	Yes/no	DASS-21	July to October 2014	NA
Li et al. (49)	China	51.8	70.7	Cross-sectional	9,371	979	Tea	Green tea: 0.97 (0.80- 1.18) Black tea: 0.39 (0.23- 0.66)	None vs. ≥3 cups/day	interview based on a self-designed questionnaire	0–≥3 cups/day	PHQ-9	NA	Age and gender, race, education level, marital status, living status, income, vegetable intake, fruits intake, red meat intake, fish intake, eggs intake, smoking, alcohol drinking, physical activity, hypertension, diabetes, coronary heart disease, Activities of Daily Living Scale scores and Mini-Mental State Examination scores.
Chanda et al. (50)	China	69.7	60–93	Cross-sectional	614	NA	Tea Coffee	Tea: 0.82 (0.71–0.95) Coffee: 0.86(0.71–1.04)	Drinking coffee or tea for Less or more than 15 years	Interviewer-administered questionnaire	Drinking coffee or tea for Less or more than 15 years	GDS-15	2011–2015	NA
Yu et al. (51)	Atlantic Canada	68.9	35–69	Cross-sectional	18,838	3,217	Coffee	Male: 1.11(0.85–1.45) Female: 1.38(1.15–1.64)	Never vs. ≥ 4 cups/day	Questionnaire	0–4 cups of coffee	PHQ-9	2009–2013	Age, ethnicity, education, province of residence, smoking status, alcohol drinking, self-reported cardiovascular disease and diabetes, healthy eating index (in tertiles), total physical activity (in MET-min/week tertiles), and BMI
Kim et al. (52)	Korea	59.7	≥19	Cross-sectional	9,576	1,443	Green tea Coffee Caffeine	Green tea: 0.79 (0.63–0.99) Coffee: 0.68 (0.55–0.85) Caffeine: 0.76(0.62–0.92)	Green tea never vs. ≥3 Cups/Week Coffee: never vs. ≥2 cups/day Caffeine: ≤22 mg/day vs. >122.9 mg/day	FFQ	Green tea: 0–≥3 Cups/Week Coffee: 0–≥2 cups/day Caffeine: ≤22–>122.9 mg/day	Assessed by some questions	NA	Adjusted for age and sex, BMI, income level, education level, alcohol intake, smoking status, physical activity, intake of energy, vegetable, fruit, red meat, fish, and green tea (or coffee)
Navarro et al. (53)	Navara	60	36.4 years	Cohort study	14,413	199	Coffee	0.37 (0.15–0.95)	<1 vs. ≥4 cups/day	FFQ	<1–≥4 cups/day	two criteria simultaneously: (a) validated physician-diagnosed depression together with (b) new onset of habitual antidepressant use	10 years	Adjusted for sex, alcohol intake (linear and quadratic term), years of university education, marital status, smoking, body mass index, total energy intake, adherence to the Mediterranean diet, between-meal snacking and following special diets, leisure-time physical activity (METS-h/week), hours of TV watching, hypertension at baseline, baseline high blood cholesterol, self-perception of competitiveness, anxiety, and psychological dependence, and use of anxiolytics, and stratified for age (decades) and recruitment period
Pogoda et al. (54)	USA	50	47.3	Cross-sectional	1,342	132	Caffeine	1.40 (0.63- 3.11)	First vs. forth quartile	24- h recall	First-forth quartile	PHQ-9	2009–2010	Adjusted for gender, race/ethnicity, smoking status, and use of antidepressants.
Iranpour et al. (55)	USA	52.8	Aged ≥18	Cross-sectional	4,737	305	Caffeine	0.23 (0.06–0.8)	First vs. forth quartile	Dietary recall	First-forth quartile	PHQ-9	2005–2006	Age, sex, family PIR, education, marital status, disease history, sleep disorders, thyroid problems, physical activity, social support, smoking, total energy, cholesterol, retinol, vitamin A, beta-carotene, beta-cryptoxanthin, vitamin B1, iron, and phosphorus levels
Kimura et al. (56)	Japan	100	65–94	A multi-center cross-sectional study	1,992	NA	Coffee Green tea Caffeine	Coffee: 0.64 (0.46–0.88) Caffeine: 0.75 (0.55–1.02) Green tea: 0.85 (0.62–1.17)	Coffee: 0–3 vs. 107–619 g/1,000 kcal Green tea: 0–99 vs. 320–788 g/1,000 kcal Caffeine: 0–119.2 vs. 234.9–758 mg/1,000 kcal	BDHQ	Coffee: 0–619 g/1,000 kcal green tea: 0–788 g/1,000 kcal) caffeine: 0–758 mg/1,000 kcal	CES-D	2011–2012	Adjusted for age, residential block, living status(alone or not alone), current smoking (yes or no), alcohol drinking (yes or no), marital status(married or nit married), physical activity level (total metabolic equivalents-hour/day: METs), size of residential area (city with a population ≥1 million, a city with a population, BMI and education(junior high school, high school, junior college, and university and higher), EPA+DHA intake (mg/1000 kcal), folate intake(mcg/1000 kcal) dietary supplement (yes/no)
Ángeles Pérez-Ara et al. (57)	Netherlands, United Kingdom, Germany, and Spain	75.3	18–75	Cross-sectional	941	312	Coffee Tea	Coffee: 1.00 (0.60–1.65) Tea: 1.10 (0.63–1.92)	Coffee: <1 cup/d vs. > 3 cups/d Tea: <1 cup/d vs. > 3 cups/d	FFQ	<1 cup/d–>3 cups/d	30-item self-administered questionnaire	September 2015 and October 2016.	Adjusted for the site, age, gender, marital status, level of education, BMI, MooDFOOD diet score, smoking, alcohol use, physical activity, high blood pressure, diabetes, and stomach or intestinal ulcer
Ng et al. (58)	Singapore	NA	Mean age 67 years	Prospective cohort study	3,177	57	Tea	0.34 (0.13- 0.90)	None or <1 cup/d vs. ≥3 cups/d	Reported habitual intake of common tea types using indigenous references	None–≥3 cups/d	GDS-15	Four years	Age, sex, ethnicity, education, housing type, single/divorced/widowed, living alone, physical and social activity, smoking, alcohol, number of comorbidities, MMSE, and baseline GDS level
Kromhout et al. (59)	Netherlands	59	82 years +9	Cross-sectional	206	145	Caffeine	0.6 (0.2–2.1)	High vs. low	Cups of coffee, tea, and cola consumed were observed and recorded six times a day.	Low-normal-high	MDS-DRS	NA	Age, gender, and stage of cognitive decline together with any of the following variables that were significantly related to the specific outcome (the use of psychotropic medication, marital status, Barthel Index total score, the presence of pain, cohort, and kidney function)
Safarini et al. (60)	Palestine	61.2	NA	Cross-sectional	1,051	598	Caffeine	Coffee: 0.573 (0.261–1.255) Tea: 0.567 (0.270–1.189)	NA	Questionnaire	NA	BDI-II	October 2020 and January 2021	Study year, gender, and academic field
Yao et al. (61)	China	54.2%	83.7	Cross-sectional	13,115	NA	Green tea	0.85 (0.76–0.95)	Never or <1 cup/month vs. ≥1 Cup/daily	Self-reported	Never or <1 cup/month <1 cup/day but ≥1 Cup/month ≥1 Cup/daily	CES-D-10	NA	The demographic factors included age and sex. Socioeconomic conditions included education, socioeconomic status, rural residence, and geographical regions. Family/social support included marital status and living arrangements. Health behaviors included social and leisure activity index, smoking, alcohol drinking, BMI (as a proxy for unhealthy behaviors), and regular dietary (vegetable/fruit/fish/nut) intake. Health status were measured by self-rated health, of 13 cognitive impairment, medical illness, comorbidity, and disability in activities of daily living (ADL)

BMI, Body Mass Index; BDI, Beck Depression Inventory; GDS, Geriatric Depression Scale; CES-D, Center for Epidemiological Studies Depression Scale; PHQ-9, Patient Health Questionnaire; HADS, Hospital Anxiety and Depression scale; DASS, Depression Anxiety Stress Scale; MDS-DRS, Minimum Data Set-based Depression Rating Scale; MINI, Mini International Neuropsychiatric Interview; ICD, International Classification of Diseases; DSM, Diagnostic and Statistical Manual of Mental Disorders; FFQ, Food Frequency Questionnaire; BDHQ, Brief Dietary History Questionnaire.

Due to confounding, four studies were rated to have a moderate risk of bias (36, 46, 52, 53), while the others were rated to have a serious risk of bias. Regarding the exposure assessment, 11 studies were classified as moderate risk (37, 43, 45, 46, 52, 53, 56, 57, 59, 62, 65), and others presented a serious risk of bias. For the selection of participants, seven studies were rated as having a moderate risk (43, 48, 53, 56, 57, 62, 65), two studies were rated as having a serious risk (59, 60), and others were rated as having a low risk of bias. Considering misclassifications during follow-up, almost all studies were classified as having a moderate risk of bias, while three studies were at low risk (47, 58, 65). Due to missing data, a moderate risk of bias was assessed in six studies (46, 47, 52, 53, 58, 65) and a low risk in the remaining studies. Two studies had serious outcome measurement bias (52, 65), while other studies were at moderate risk. All 29 studies were judged to be at low risk of bias due to selective reporting of results, and, ultimately, the overall judgment of two studies showed a moderate risk of bias (46, 53), while 27 were at serious risk (Table 2).

**Table 2:** ROBINS-I judgment for each domain and overall.

**Study**	**Bias due to confounding**	**Bias due to the selection of participants**	**Bias due to exposure assessment**	**Bias due to misclassification during follow-up**	**Bias due to missing data**	**Bias due to measurement of the outcome**	**Bias due to selective reporting of the results**	**Overall judgment**
Hintikka et al. (34)	Serious	Low	Serious	Moderate	Low	Moderate	Low	Serious
Kendler et al. (35)	Serious	Low	Serious	Moderate	Low	Moderate	Low	Serious
Niu et al. (36)	Moderate	Low	Serious	Moderate	Low	Moderate	Low	Serious
Ruusunen et al. (37)	Serious	Low	Moderate	Moderate	Low	Moderate	Low	Serious
Smith (38)	Serious	Low	Serious	Moderate	Low	Moderate	Low	Serious
Chen et al. (39)	Serious	Low	Serious	Moderate	Low	Moderate	Low	Serious
Lucas et al. (40)	Serious	Moderate	Moderate	Low	Moderate	Serious	Low	Serious
Feng et al. (41)	Serious	Low	Serious	Moderate	No information	Moderate	Low	Serious
Feng et al. (42)	Serious	Low	Serious	Moderate	Low	Moderate	Low	Serious
Pham et al. (43)	Serious	Moderate	Moderate	Moderate	Low	Moderate	Low	Serious
Tsai et al. (44)	Serious	Low	Serious	Moderate	Low	Moderate	Low	Serious
Park and Moon (45)	Serious	Low	Moderate	Moderate	Low	Moderate	Low	Serious
Omagari et al. (36)	Serious	Moderate	Moderate	Moderate	Low	Moderate	Low	Serious
Guo et al. (46)	Moderate	Low	Moderate	Moderate	Moderate	Moderate	Low	Moderate
Ritchie et al. (47)	Serious	Low	Serious	Low	Moderate	Moderate	Low	Serious
Taher et al. (48)	Serious	Moderate	Serious	Moderate	Low	Moderate	Low	Serious
Li et al. (49)	Serious	Low	Serious	Moderate	Low	Moderate	Low	Serious
Chanda et al. (50)	Serious	Low	Serious	Moderate	Low	Moderate	Low	Serious
Yu et al. (51)	Serious	Low	Serious	Moderate	No information	Moderate	Low	Serious
Kim et al. (52)	Moderate	Low	Moderate	Moderate	Moderate	Serious	Low	Serious
Navarro et al. (53)	Moderate	Moderate	Moderate	Moderate	Moderate	Moderate	Low	Moderate
Pogoda et al. (54)	Serious	Low	Serious	Moderate	Low	Moderate	Low	Serious
Iranpour et al. (55)	Serious	Low	Serious	Moderate	Low	Moderate	Low	Serious
Kimura et al. (56)	Serious	Moderate	Moderate	Moderate	Low	Moderate	Low	Serious
Ángeles Pérez-Ara et al. (57)	Serious	Moderate	Moderate	Moderate	Low	Moderate	Low	Serious
Ng et al. (58)	Serious	Low	Serious	Low	Moderate	Moderate	Low	Serious
Kromhout et al. (59)	Serious	Serious	Moderate	Moderate	Low	Moderate	Low	Serious
Safarini et al. (60)	Serious	Serious	Serious	Moderate	Low	Moderate	Low	Serious
Yao et al. (61)	Serious	Low	Serious	Moderate	Low	Moderate	Low	Serious

### 3.2. Association of coffee consumption with depression

**Figure 1:**
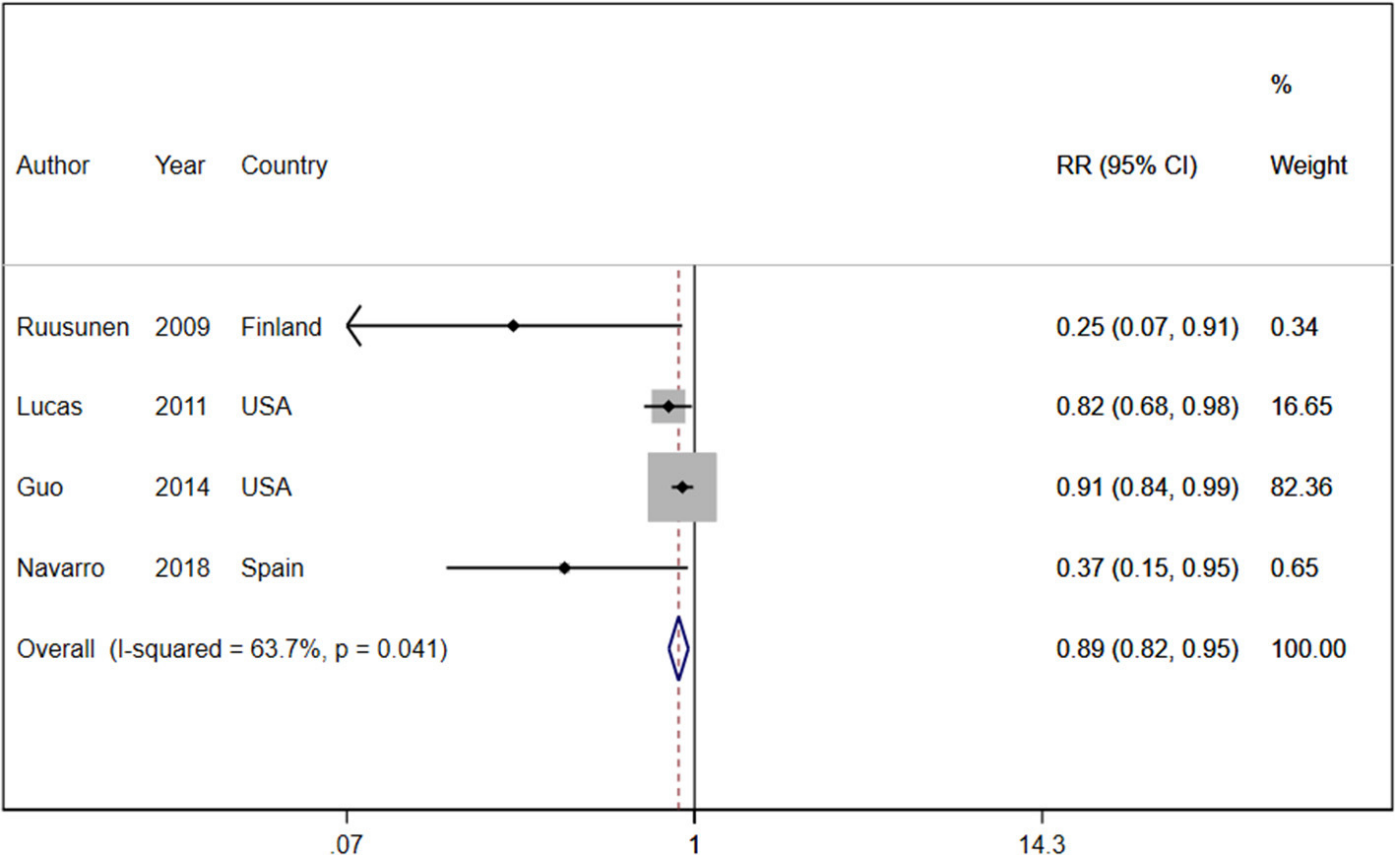
Relative risk of depressive symptoms for the highest compared with the lowest category of coffee intake. RR, relative risk.

**Figure 2:**
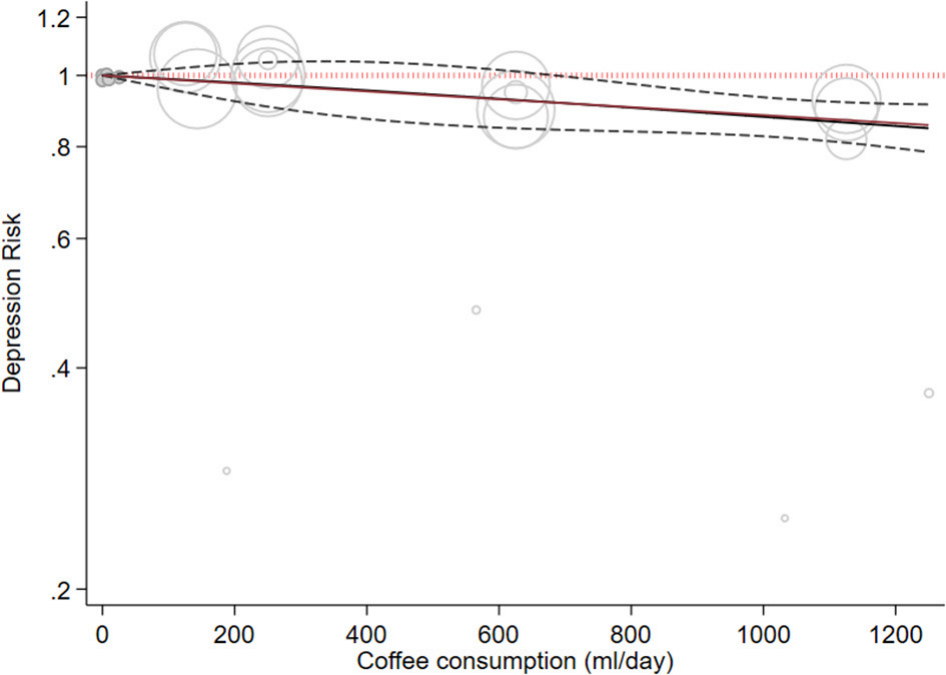
Dose-response association between coffee intake and depressive symptoms. The solid line represents a non-linear dose response, and the dotted lines represent a 95% confidence interval. Circles represent hazard ratio point estimates for coffee intake categories from each study with circle size proportional to the inverse of standard error.

Four cohort studies with a total of 319,996 participants and 13,583 cases reported information about coffee consumption and depressive symptoms (37, 46, 53, 65). Comparing the highest with the lowest category, there was an inverse association between intake of coffee and depressive symptoms (RR:0.89, 95% CI: 0.82–0.95; I^2^ = 63.7%, *P*_heterogeneity_ = 0.04; Figure 1). Four studies were eligible for the linear dose-response analysis (37, 46, 53, 65). An increase in coffee intake of 240 ml per day was associated with a 4% lower risk of developing depression (RR: 0.96, 95% CI: 0.95, 0.98; I^2^ = 22.7%, *P*_dose−response_ < 0.001) (Figure 2). We did not find a nonlinear association between coffee intake and depression risk (*P*_non − linearity_ = 0.89) (Figure 2). Our subgroup analyses suggested that there were potential sources of heterogeneity based on geographical region, follow-up duration, and the number of participants (Supplementary Table 3). Based on a visual inspection of the funnel plot, we found some asymmetry (Supplementary Figure 2). Egger's regression test indicated possible publication bias (*P* = 0.001). Findings from other sensitivity analyses revealed that excluding any single study from the analysis did not appreciably alter the pooled effect sizes.

Ten cross-sectional studies with a total of 45,883 participants reported information about coffee consumption and depressive symptoms (34, 36, 43, 45, 50, 51, 57, 62, 66, 67). Comparing the highest with the lowest category, coffee intake had an inverse association with depressive symptoms (RR: 0.78, 95% CI: 0.62, 0.98; I^2^ = 81.3%, *P*_heterogeneity_ = 0.001; Supplementary Figure 3). Our subgroup analyses showed heterogeneity based on geographical region, smoking status, physical activity, energy intake, alcohol consumption, and BMI (Supplementary Table 4).

### 3.3. Association of tea consumption with depression

**Figure 3:**
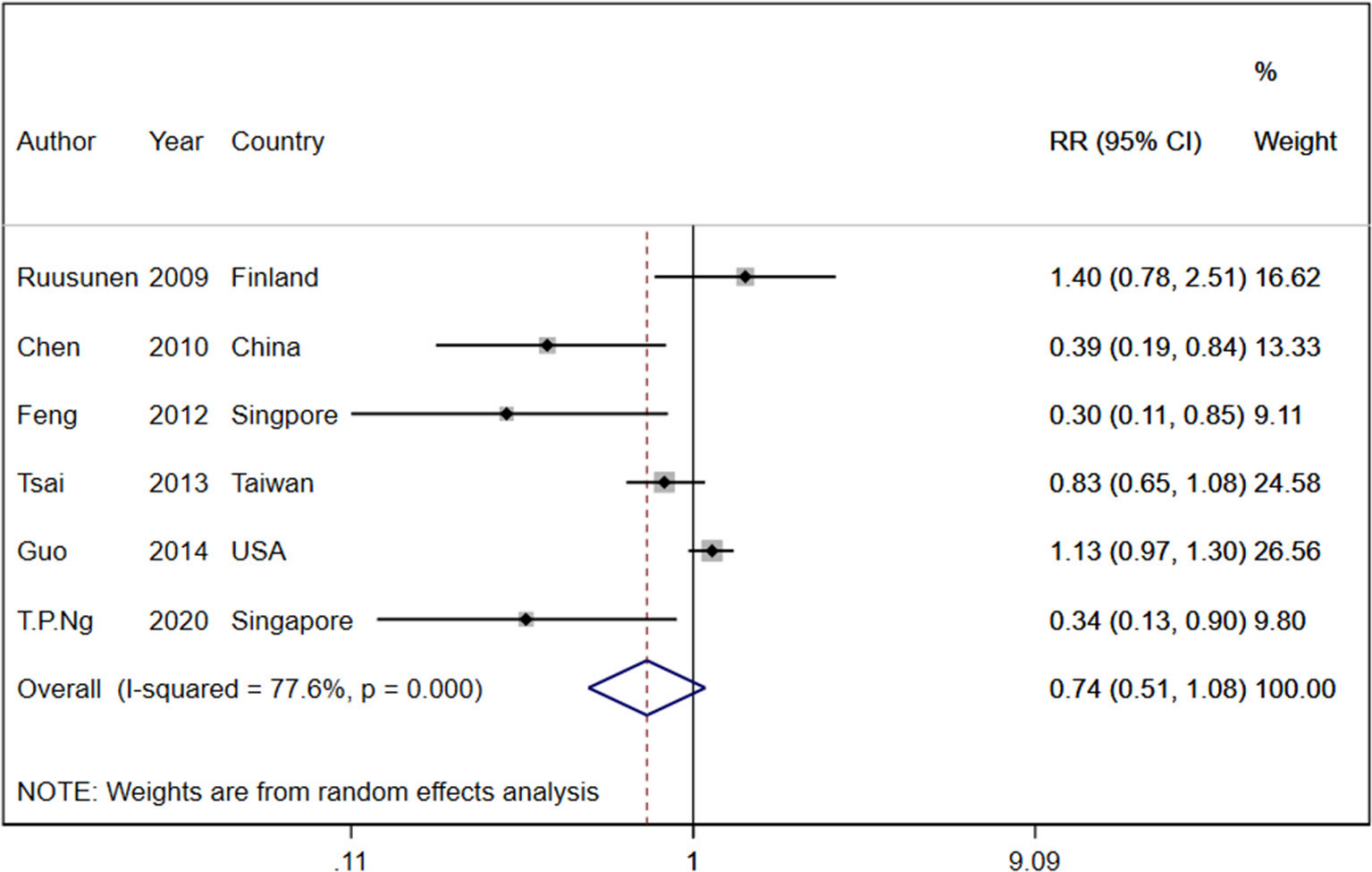
Relative risk of depressive symptoms for the highest compared with the lowest category of tea intake. RR, relative risk.

**Figure 4:**
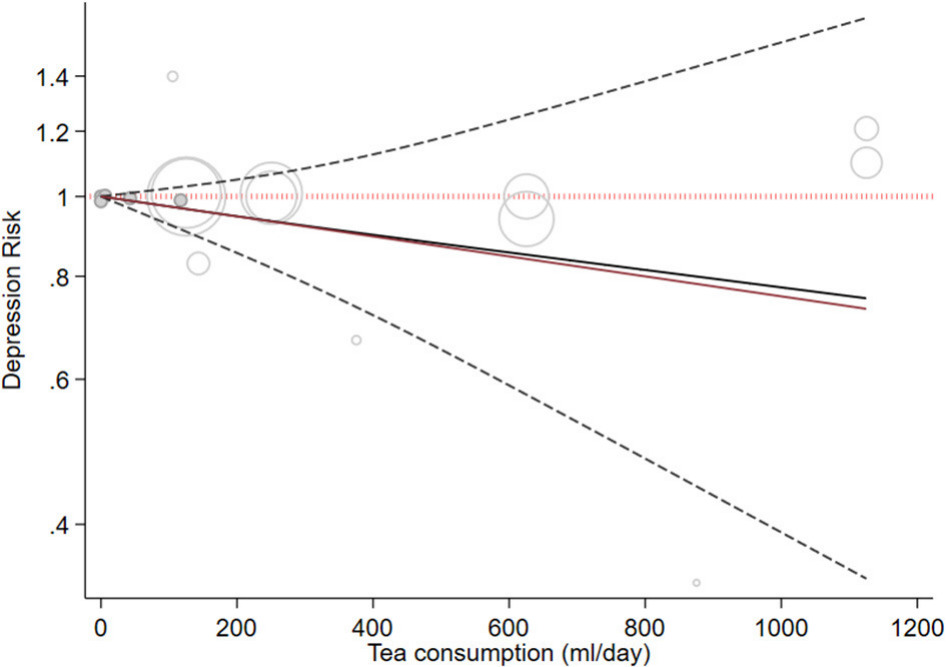
Dose-response association between tea intake and depressive symptoms. The solid line represents a non-linear dose response, and the dotted lines represent a 95% confidence interval. Circles represent hazard ratio point estimates for coffee intake categories from each study with circle size proportional to the inverse of standard error.

Six cohort studies with a total of 263,180 participants and 12,471 cases reported information about tea consumption and depressive symptoms (37, 39, 41, 44, 46, 58). On comparing the highest category with the lowest category in random effects analysis, there was no significant inverse association between tea consumption and depressive symptoms (RR: 0.74, 95%CI: 0.51, 1.08; I^2^ = 77.6%, *P*_heterogeneity_ = 0.001; Figure 3). In the linear dose-response analysis of tea consumption and depression risk, based on four cohort studies, an increase in tea intake of 240 ml/day was not associated with the risk of depression (RR: 0.99, 95%CI: 0.97, 1.01; I^2^ = 58%). We did not find a dose-response association between tea consumption and the risk of depression (*P*_dose − response_ = 0.405) (Figure 4). There was no non-linear association between tea intake and the risk of depression (*P*_non − linearity_ = 0.89) (Figure 4). Our subgroup analyses showed heterogeneity based on the BMI, alcohol consumption, geographical region, follow-up duration, number of participants, and smoking status. The subgroup analysis indicated a significant inverse association in studies conducted in Asia (RR: 0.73, 95%CI: 0.58, 0.92; I^2^ = 67%, *n* = 3) as well as a significant inverse association in studies that did not adjust for alcohol intake (RR: 0.77, 95%CI: 0.60, 0.98; I^2^ = 71%, *n* = 2), BMI (RR: 0.73, 95%CI: 0.58, 0.92; I^2^ = 67%, *n* = 3), or smoking (RR: 0.77, 95%CI: 0.60, 0.98; I^2^ = 71%, *n* = 2) (Supplementary Table 5). Based on the visual inspection of the funnel plot, we found asymmetry (Supplementary Figure 4); however, when we did Egger's regression tests, no significant publication bias was observed (*P* = 0.07). Upon excluding the study by Ruusunen et al. (37) from the analysis, there was a significant negative association between tea consumption and depression (RR: 0.64, 95% CI: 0.41–0.99).

Twelve cross-sectional studies with a total of 47,882 participants reported information about tea consumption and depressive symptoms (34, 36, 42–44, 49, 50, 52, 56–58, 61). On comparing the highest category with the lowest category, total tea, green tea, and black tea consumption had an inverse association with depressive symptoms (RR:0.73, 95%CI: 0.65, 0.81; I^2^ = 61.0%, *P*_heterogeneity_ = 0.025, RR:0.84, 95%CI: 0.78, 0.90; I^2^ = 45.3%, *P*_heterogeneity_ = 0.103, RR:0.58, 95%CI: 0.43, 0.79; I^2^ = 70.1%, *P*_heterogeneity_ = 0.067; respectively Supplementary Figure 5). Our subgroup analyses showed heterogeneity based on the number of participants (Supplementary Table 6).

### 3.4. Association of caffeine consumption with depression

**Figure 5:**
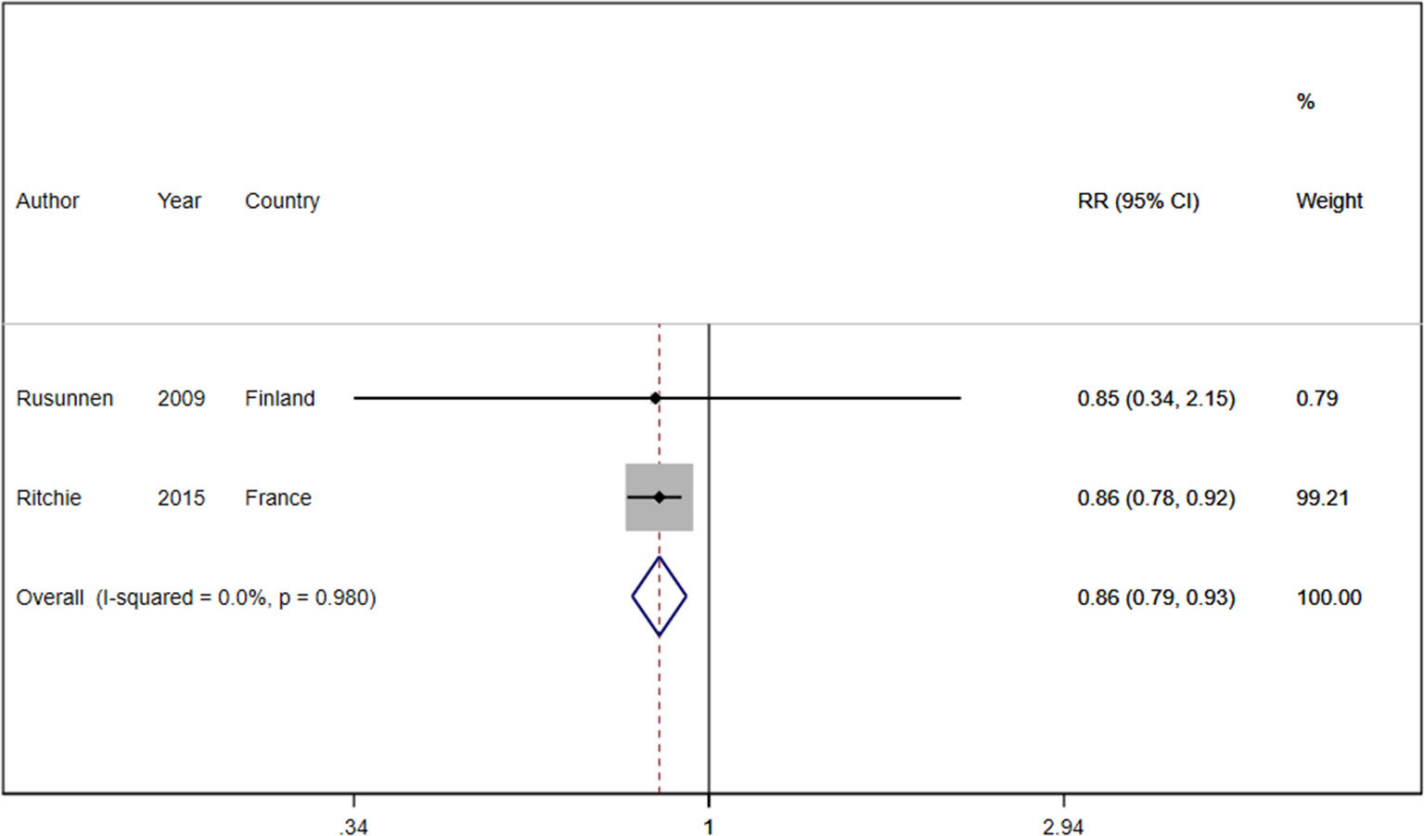
Relative risk of depressive symptoms for the highest compared with the lowest category of caffeine intake. RR, relative risk.

**Figure 6:**
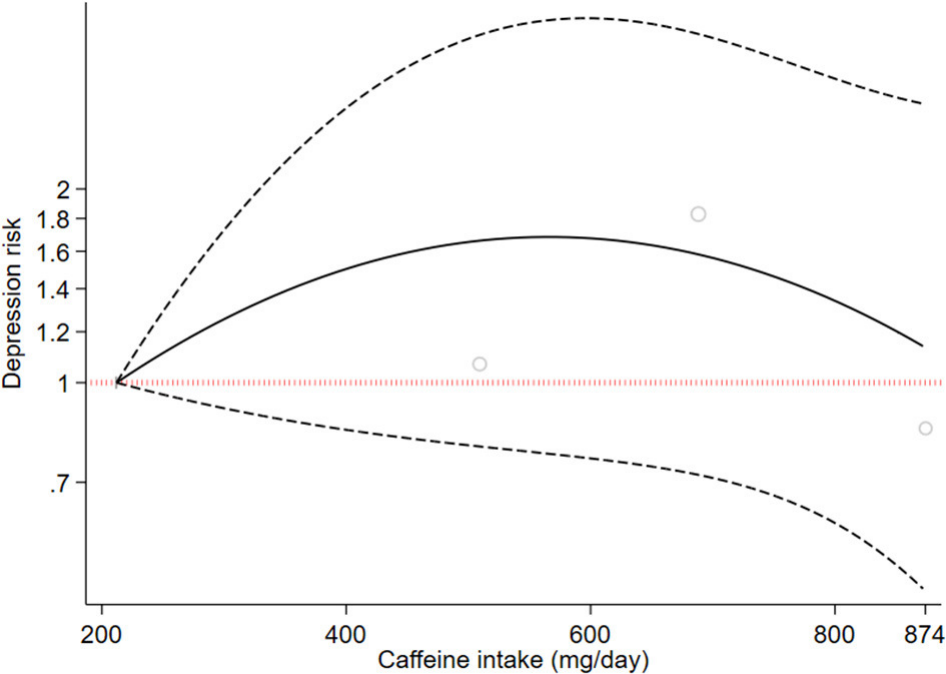
Dose-response association between caffeine intake and depressive symptoms. The solid line represents a non-linear dose response, and the dotted lines represent a 95% confidence interval. Circles represent hazard ratio point estimates for coffee intake categories from each study with circle size proportional to the inverse of standard error.

Two cohort studies with a total of 8,017 participants and 1,125 cases reported information about caffeine intake and depressive symptoms (37, 47). On comparing the highest category with the lowest category, caffeine intake was inversely associated with depressive symptoms (RR: 0.86, 95%CI: 0.79, 0.93; I^2^ = 0.0%, *P*_heterogeneity_ = 0.980; Figure 5). One cohort study was eligible for dose-response analysis (37). An increase in caffeine intake of 200 mg/day was not associated with the risk of depression (RR: 1.05, 95%CI: 0.80, 1.36; I^2^ = 0%). A non-linear dose-response meta-analysis performed on one cohort study indicated a non-statistically significant increasing association with intake of 200–600 mg of caffeine (*P*_dose − response_ = 0.163 Figure 6) and an inverse association with higher intake (*P*_dose − response_ = 0.18, Figure 6); however, there was a nonlinear association (*P*_non − linearity_ = 0.01 Figure 6).

Ten cross-sectional studies with a total of 34,495 participants reported information about caffeine intake and depressive symptoms (35, 38, 43, 47, 52, 54–56, 59). Comparing the highest with the lowest category, caffeine intake did not have an inverse association with depressive symptoms (RR: 0.64, 95% CI: 0.40, 1.01; I^2^ = 95.3%, *P*_heterogeneity_ < 0.001; Supplementary Figure 6). Based on a visual inspection of the funnel plot, we found some asymmetry (Supplementary Figure 7); however, when we did Egger's regression tests, no significant publication bias was observed (*P* = 0.34). The overall effect size depended on the studies of Kendler et al. (35) and Pogoda et al. (54). By excluding these studies, a significant negative association was found between caffeine and depression risk (RR: 0.55, 95% CI: 0.34–0.88, RR: 0.58, 95%CI: 0.36–0.95, respectively).

### 3.5. Grading the evidence

We applied the GRADE tool to make a judgment on the quality of the evidence. The quality of evidence was rated low for coffee intake due to devaluations for risk of bias and inconsistency, very low for tea intake due to downgrades for risk of bias, inconsistency, and imprecision, and moderate for dietary caffeine intake associated with downgrades for risk of bias (Supplementary Table 7).

## 4. Discussion

Our systematic review and dose-response meta-analysis of observational studies revealed that a higher intake of caffeine, coffee, and tea was inversely associated with the risk of depressive symptoms. Higher coffee consumption was associated with an 11 and 22% lower risk of depressive symptoms in the cohort and cross-sectional studies, respectively, and there was also a significant linear dose-response relationship. When we compared higher tea consumption to lower consumption in cohort studies, we found that the risk of depressive symptoms was reduced by 26%, and this inverse association was replicated in cross-sectional studies; however, we did not find a dose-response association. An analysis of cohort and cross-sectional study results investigating the association between caffeine intake and the risk of depressive symptoms found that a higher caffeine intake was associated with a 14 and 13% lower risk of depressive symptoms, respectively.

These results provide supportive evidence consistent with the findings of previous meta-analyses of observational studies examining the association between coffee, tea consumption, and depression. Grosso et al. suggested that coffee and tea consumption act as protective factors for depression. It was noted that the caffeine content may account for some of the beneficial effects, but this does not fully explain the established relationship (68). Kang et al. also showed quantitative evidence for the inverse association between high coffee and tea consumption and the risk of developing depression but concluded that it is difficult to determine the causality of this association because of the observational design of the studies and insufficient prospective studies for this topic (69). Regarding the association between coffee and caffeine consumption and depression, Wang et al. conducted a meta-analysis of observational studies and also identified a strong inverse association (70). Dong et al. also performed a meta-analysis of observational studies focusing on the relationship between tea consumption and depression, which led to the identification of tea as a potential preventive factor against depression (71). A recent study by Fisicaro et al. (72) found that higher mocha coffee consumption was associated with a higher level of psycho-cognitive functioning in elderly non-smokers in Italy with chronic subcortical ischaemic vascular disease (SIVD) and cognitive profile of mild vascular cognitive impairment (mVCI). This finding is consistent with previous findings and further supports the fact that coffee has positive effects on the cognitive process as well (72).

Moreover, the results of some studies are inconsistent with the results of the present meta-analysis. In the study conducted by Kendler et al., it was shown that caffeine consumption by people who are highly vulnerable to caffeine dependence may make them more prone to chronic symptoms of anxiety and depression (35). A meta-analysis by Pogoda et al. did not report caffeine to be protective against depression (54). Jee et al. (73) showed that the beneficial and/or harmful effects of caffeine on several neurological and psychiatric disorders may differ depending on gender. Women may be more likely to experience a reduction in stroke risk with caffeine consumption. However, caffeine consumption may also increase the risk of sleep disorders for both sexes equally. A greater reduction in dementia risk was also observed in women than in men, according to a review. However, caffeine had a greater protective effect against Parkinson's disease in men than in women (73). A cohort study on 3,323 students aged 11 to 17 years found that the impact of caffeine on anxiety varied between sexes, with no significant effects observed in girls, while in boys, caffeine consumption was associated with an increase in anxiety levels (73, 74). Different kinds of disease, different disease types, and different dosages of caffeine or different kinds of tea or coffee may be the reasons for these contradictions. Therefore, further research is needed to expand the existing knowledge in this field.

The mechanisms underlying the inverse association between the consumption of caffeine (coffee and tea) and the risk of developing depression are not yet fully determined; however, some possible biological explanations have been suggested. The first favorable effect can be mediated by caffeine. Caffeine stimulates the central nervous system since it is a non-specific adenosine A1/A2A receptor antagonist, generating psychostimulant effects through modulating dopaminergic transmission by increasing calcium signaling (15, 75, 76). Caffeine metabolites have an effect on adenosine receptors in the brain, which helps to alleviate depression (76). The second important mechanism that had been suggested to play a protective role against depression is related to a complex mixture of chemicals with anti-inflammatory activities. It is common knowledge that the pathophysiology of depression is correlated with low-grade inflammation and oxidative stress dysregulation (9, 77–79). Specific phenolic compounds in coffee, called chlorogenic acid and caffeic acid, play an anti-inflammatory and antioxidant role, leading to the reduction of oxidative stress (Supplementary Figure 8) (80–82).

Tea shares similar effects as it is the main source of polyphenols, particularly catechins. Epigallocatechin gallate (EGCG) is known to be the most potent tea component, counteracting depression through powerful antioxidant activity (17). Another antidepressant measure taken by EGCG is monoamine oxidase (MAO) inhibition, leading to a higher dopamine and serotonin concentration in the brain (83, 84). In addition to the properties mentioned, some studies demonstrated a probable antidepressant effect of EGCG *via* an increase in brain-derived neurotrophic factor, neuronal survival, and plasticity (83). The benefits of tea are not only related to its caffeine and polyphenol content. Theanine, the main amino acid found in the tea has beneficial effects against depression by increasing serotonin and dopamine in the brain, which are considered to be two key neurotransmitters in the etiology of depression (Supplementary Figure 8) (18, 19).

The strengths of our study deserve consideration. First, we applied the best-fitting second-order fractional polynomial to model curvilinear associations when restricted cubic splines could not be calculated because of the limited number of studies (*n* ≤ 2) included in the analyses. Second, we also considered all studies carried out from 2015 to 2021, including 12 cross-sectional studies and two prospective cohort studies in our analysis, which were not considered in previous systematic reviews and meta-analyses. Finally, the GRADE system was applied to rate the overall quality of the evidence.

Our study also has some potential limitations. The current study is based on observational studies with potentially unknown confounders that affect estimates of effects, but most studies were adjusted for potential confounders such as age, gender, health status, dietary intake, smoking status, physical activity, and socioeconomic conditions. Nonetheless, unmeasured confounders might have biased evaluations and results, such as family or social support. Due to the cross-sectional design, we cannot ensure whether caffeine, coffee, and tea consumption have reduced the risk of developing depression or whether individuals with depression symptoms consume less than non-depressed individuals. Various methods have been used to measure caffeine, tea, and coffee consumption (e.g., grams, cups, and times). Regarding depression assessment, a variety of instruments were used, from specific questionnaires to patient reports.

## 5. Conclusion

In summary, our results suggest that consuming coffee, tea, and dietary caffeine may have protective effects against depression and may lower the risk of developing depression. However, it is crucial to conduct well-designed prospective studies using harmonized tools in the future for both exposure and outcome assessments.

## Data availability statement

The data analyzed in this study is subject to the following licenses/restrictions: The datasets generated or analyzed during the current study are not publicly available but are available from the corresponding author on reasonable request. Requests to access these datasets should be directed to SJ, jazayeri.sh@iums.ac.ir.

## Ethics statement

This research was conducted in accordance with the Research Ethics Committees of the Iran University of Medical Sciences (Ethics No. IR.IUMS.REC.1401.814).

## Author contributions

KT and HS designed the project, performed the literature search, and wrote the first draft of the manuscript. HS analyzed the data and interpreted the results. KT, NP, and SJ revised the subsequent drafts for important intellectual content. SJ was the guarantor. All authors read and approved the final version of the manuscript.
