# Supplementary material for: Association between dietary caffeine, coffee, and tea consumption and depressive symptoms in adults: A systematic review and dose-response meta-analysis of observational studies
Source: Front Nutr. 2023 Feb 9;10:1051444. doi: 10.3389/fnut.2023.1051444 (PMC9947483; doi:10.3389/fnut.2023.1051444)
Supplement: Supplementary file 1 [file Data_Sheet_1.docx]

Supplementary Materials including Supplementary Tables 1-7 and Supplementary Figures 1-8

**Supplementary Table 1.** Search strategy to find potential eligible cohort studies.

| 1. “Coffee” OR “Tea” “Green tea” OR “Black tea” OR “Coffea” OR “caffeine” OR “beverages” |
| --- |
| 2. “Depress*” OR “Depression” OR “Depressive” OR “Depressed” OR “Depressive symptom” OR “Emotional depression” OR “Depression disorder” OR “Depression risk” |
| 3. ((("Observational Study" [Publication Type] OR "Observational Studies as Topic"[Mesh]) OR ("Longitudinal Studies"[Mesh] OR "Prospective Studies"[Mesh])) OR ("Cohort Studies"[Mesh])) OR (((((((((((((cohort*) OR (prospective*)) OR (retrospective*)) OR (longitudinal)) OR (observational)) OR (follow-up)) OR (nested case-control)) OR (case-cohort)) OR ("case control")) OR ("relative risk")) OR ("hazard ratio")) OR ("odds ratio")) OR (risk)) |
| 4. # 1 AND #2 AND #3 |

**Supplementary Table 2**: Description and decision criteria for each domain in ROBINS-I

| **Domain** | **Explanation** | **Judgments** |
| --- | --- | --- |
| **Bias due to confounding** | - Is there potential for confounding of the effect of exposure in this study? - Did the authors use a multivariable-adjusted analysis method that controlled at least for age, sex, smoking, physical activity, and body mass index? - Did the authors avoid adjusting for post-exposure variables?   Notes: Confounding is expected in all observational studies; a low risk of bias was not assigned to any study. Time-varying confounding was expected to be unlikely and is not expected to cause a risk of bias in the present study. | Low risk of bias: No bias expected due to confounding, including time-varying confounding.  Moderate risk of bias: Confounding is expected: age, sex, smoking, physical activity, and body mass index have been appropriately controlled for in a multivariable-adjusted analysis  Serious risk of bias: At least one known important domain was not measured or appropriately controlled for  No information: No information on which confounders have been controlled for. |
| **Bias due to selection of participants** | - Was the selection of participants into the study based on participant’s characteristics observed after the start of the study/exposure assessment? - Do the start of follow-up and the start of exposure coincide for most participants? Were methods used that are likely to correct for the presence of selection biases? | Low risk of bias: All participants who would have been eligible for the target study were included in the study; *and* the authors conducted a sensitivity analysis excluding T2D cases which occurred <2 years after start of the study and the results did not change.  Moderate risk of bias: Selection into the study may have been related to exposure and outcome (e.g., inclusion of postmenopausal women only); *and* the authors used appropriate methods to correct for the selection bias;  *or* the authors conducted no sensitivity analysis excluding T2D cases which occurred <2 years after the start.  Serious risk of bias: Selection into the study was related to exposure and outcome (e.g., only participants with prediabetes were included in the analysis); *and* this could not be corrected for in the analyses;  *or* the start of follow-up and start of exposure do not coincide *and* the rate ratio is not constant over time.  No information: No information is reported about selection of participants into the study. |
| **Bias due to exposure assessment** | - Were exposure groups clearly defined and adequately assessed? - Was the information used to define the exposure groups based on reasonable a priori data? | Low risk of bias: Exposure status was well defined (comprehensibly derived categories); *and* no measurement error is expected in its assessment.  Moderate risk of bias: Exposure status is well defined (comprehensibly derived categories); *and* exposure was measured using a validated tool (e.g. a validated FFQ).  Serious risk of bias: Exposure status is not well defined; *and* exposure was measured using not validated tools.  No information: No definition of exposure or no explanation of the source of information about exposure status is reported. |
| **Bias due to misclassification during follow-up** | - Were there deviations from the exposure beyond what would be expected in usual practice? - Were these deviations unbalanced between groups and likely to have affected the outcome? | Low risk of bias: Repeated measurements of the exposure status during follow-up are available. No or only slight changes in fat intake were observed and the changes were considered in the analysis.  Moderate risk of bias: Repeated measurements of the exposure are not available, but high changes are not expected during follow-up (compare notes)  or repeated measurements of the exposure status during follow-up are available and some changes in lifestyle factors were observed. The analysis was appropriate to estimate the effect of changes in lifestyle factors, allowing for deviations that were likely to impact the outcome;  Serious risk of bias: Exposure status is measured during follow-up and high changes in lifestyle factors have been observed, and the analysis was not appropriate to estimate the effect of changes in lifestyle factors, allowing for deviations that were likely to impact the outcome.  No information: No information on deviations from the exposure is reported. |
| **Bias due to missing data** | - Were there missing outcome data? - Were participants excluded due to missing data on exposure status? - Were participants excluded due to missing data on other variables needed for analysis? | Low risk of bias: Little loss-to-follow-up (<20%) and data on exposure and other variables were reasonably complete (<10% missing data) and was unlikely to introduce bias;  *or* the analysis addressed missing data and is likely to have removed any risk of bias.  Moderate risk of bias: There is a proportion of missing data in the original cohort or a high proportion of loss-to-follow-up; *and* the analysis is unlikely to have removed the risk of bias arising from the missing data (e.g., using logistic regression).  Serious risk of bias: High proportions (>50%) of missing data; *and* the analysis is unlikely to have removed the risk of bias arising from the missing data;  *or* missing data were addressed inappropriately in the analysis;  *or* the nature of the missing data means that the risk of bias cannot be removed through appropriate analysis.  No information: No information is reported about missing data or the potential for data to be missing. |
| **Bias due to measurement of the outcome** | - Could the outcome measure have been influenced by knowledge of the exposure status? - Were the methods of outcome assessment comparable across exposure groups? - Was any systematic error in the measurement of the outcome related to exposure status? | Low risk of bias: The methods of outcome assessment were comparable across exposure groups; *and* the outcome measure was unlikely to be influenced by knowledge of the exposure status of study participants; *and* any error in measuring the outcome is unrelated to exposure status (i.e., objective measures such as confirmed medical records, record linkage).  Moderate risk of bias: The methods of outcome assessment were comparable across exposure groups; *and* any error in measuring the outcome may be minimally related to exposure status *or* if the outcome measure was not reliable measured (i.e. confirmed records are not available for the whole study population).  Serious risk of bias: The methods of outcome assessment were not comparable across exposure groups;  *or* the outcome measure was subjective (i.e., self-report of type 2 diabetes by study participants); *and* error in measuring the outcome was related to exposure status.  No information: No information is reported about the methods of outcome assessment. |
| **Bias due to selective reporting of the results** | - Is the reported effect estimate likely to be selected from multiple analyses of the exposure-outcome relationship? - Is the reported effect estimate likely to be selected from different subgroups? | Low risk of bias: There is a clear description of all analyses and the analyses are consistent and all reported results correspond to all intended outcomes, analyses and sub-cohorts.  Moderate risk of bias: The analyses are clearly defined; *and* there is an indication of selection of the reported analysis from among multiple analyses; *and* there is an indication of selection of the cohort or subgroups for analysis and reporting on the basis of the results (e.g., estimates not shown for all analyses).  Serious risk of bias: There is a high risk of selective reporting from among multiple analyses; *or* the cohort or subgroup is selected from a larger study for analysis and appears to be reported based on the results.  No information: There is too little information to make a judgment. |
| **Overall judgment** | Low risk of bias | The study is judged to be at a low risk of bias for all domains. |
|  | Moderate risk of bias | The study is judged to be at low or moderate risk of bias for all domains. |
|  | Serious risk of bias | The study is judged to be at serious risk of bias in at least one domain, but not at critical risk in any domain. |

**Identification of studies via databases and registers**

Records removed *before screening*:

Duplicate records removed (n =228)

Records marked as ineligible by automation tools (n = 0)

Records removed for other reasons (n = 0)

Records identified from*:

Databases (n =1348)

PubMed (295) and Scopus (1053)

Manually (n=5)

**Identification**

Records screened

(n = 1125)

Records excluded**

(n = 1063)

Reports sought for retrieval

(n = 62)

Reports not retrieved

(n = 0)

**Screening**

Reports assessed for eligibility

(n =62)

Full-text articles excluded, with reasons
(n =33 )

Not relevant outcome = 22

Review = 11

Studies included in review

(n = 29)

Reports of included studies

(n = 29)

**Included**

**Supplementary Figure 1.** Study selection process for inclusion in the meta-analysis of coffee, tea and caffeine intake and risk of depressive symptoms.

| **Supplementary Table 3.** Subgroup analysis of coffee intake and depressive symptoms in cohort studies (highest versus lowest category meta-analysis, Relative risks and 95% confidence intervals) | | | | | | |
| --- | --- | --- | --- | --- | --- | --- |
| **Subgroup** | **N** | **RR**  **(95% CI)** | **I^2^**  **(%)** | ***P* Heterogeneity** | **P within**^†^ | **P Between**^*^ |
| **Country** |  |  |  |  |  | 0.008 |
| European | 2 | 0.32 (0.15, 0.68) | 0.0% | 0.627 | 0.003 |  |
| American | 2 | 0.89 (0.83, 0.96) | 3.7% | 0.308 | 0.003 |  |
| **Follow-up duration** |  |  |  |  |  | 0.200 |
| <11 years | 2 | 0.80 (0.67, 0.95) | 63.6% | 0.097 | 0.012 |  |
| >11 years | 2 | 1.91 (0.83, 0.98) | 74.2% | 0.0.049 | 0.017 |  |
| **Number of participants** |  |  |  |  |  | 0.233 |
| <6000 | 2 | 0.80 (0.67, 0.96) | 69.0% | 0.072 | 0.016 |  |
| >6000 | 2 | 0.90 (0.83, 0.98) | 72.4% | 0.057 | 0.015 |  |
| Abbreviations: N: number; CI, confidence interval; RR: relative risk  † P heterogeneity within subgroups.  * P for subgroup difference was calculated using Cochran’s Q test | | | | | | |


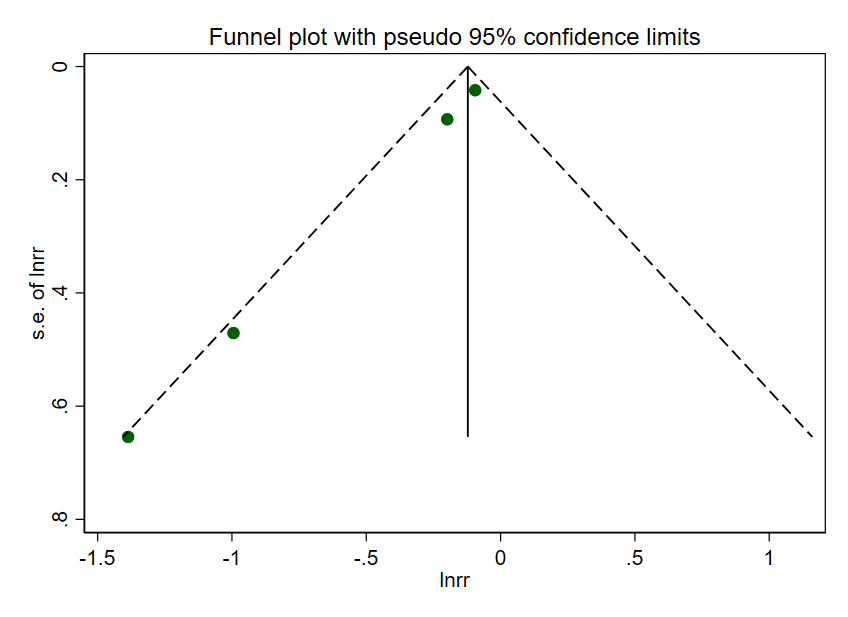


**Supplementary Figure 2.** Funnel plot of the relative risks of 4 cohort studies about coffee intake (highest versus lowest category analysis) and risk of depressive symptoms. Egger test *P*=0.001. Log RR: natural logarithm of relative risk. s.e: standard error.


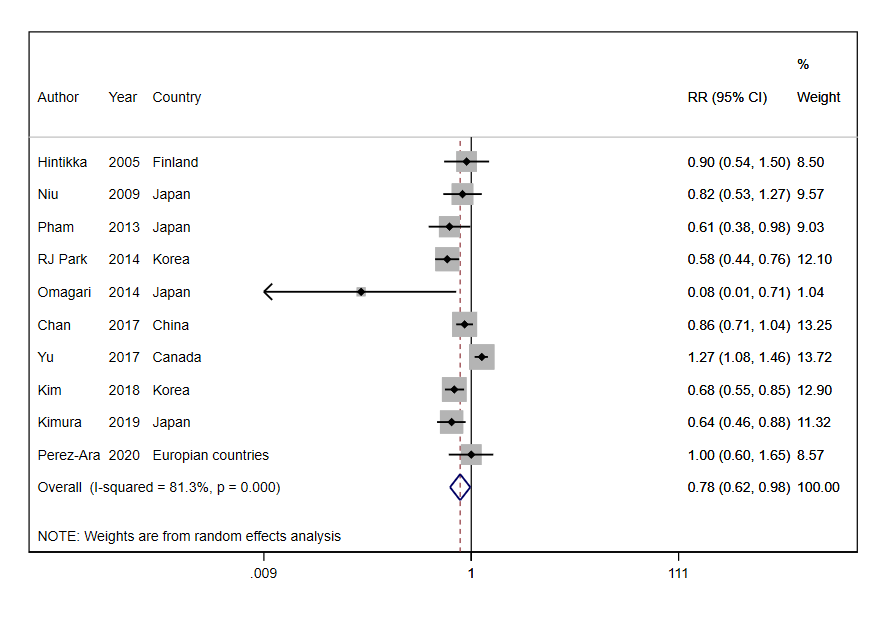


**Supplementary Figure 3.** Relative risk of depressive symptoms for the highest versus lowest category of coffee intake. RR, relative risk.

| **Supplementary Table 4.** Subgroup analysis of coffee intake and depressive symptoms in cross-sectional studies (highest versus lowest category meta-analysis, Relative risks and 95% confidence intervals) | | | | | | |
| --- | --- | --- | --- | --- | --- | --- |
| **Subgroup** | **n** | **RR**  **(95% CI)** | **I^2^**  **(%)** | ***P* Heterogeneity** | **P within**^†^ | **P Between**^*^ |
| **Country** |  |  |  |  |  | <0.001 |
| European | 3 | 1.22 (1.06, 1.40) | 10.2% | 0.32 | 0.006 |  |
| Asian | 7 | 0.71 (0.64, 0.80) | 45.9% | 0.08 | <0.001 |  |
| **Number of participants** |  |  |  |  |  | 0.055 |
| <2000 | 6 | 0.79 (0.68, 0.91) | 41.3% | 0.130 | 0.001 |  |
| >2000 | 4 | 0.94 (0.84, 1.05) | 91.6% | <0.001 | 0.251 |  |
| **Alcohol consumption** |  |  |  |  |  | 0.016 |
| Yes | 7 | 0.94 (0.85, 1.05) | 81.7% | <0.001 | 0.260 |  |
| No | 3 | 0.75 (0.64, 0.87) | 78.5% | 0.009 | <0.001 |  |
| **BMI** |  |  |  |  |  | 0.016 |
| Yes | 7 | 0.94 (0.85, 1.05) | 81.7% | <0.001 | 0.106 |  |
| No | 3 | 0.75 (0.64, 0.87) | 78.5% | 0.009 | <0.001 |  |
| **Physical activity** |  |  |  |  |  | 0.016 |
| Yes | 7 | 0.94 (0.85, 1.05) | 81.7% | <0.001 | 0.260 |  |
| No | 3 | 0.75 (0.64, 0.87) | 78.5% | 0.009 | <0.001 |  |
| **Smoking** |  |  |  |  |  | 0.016 |
| Yes | 7 | 0.94 (0.85, 1.05) | 81.7% | <0.001 | 0.260 |  |
| No | 3 | 0.75 (0.64, 0.87) | 78.5% | 0.009 | <0.001 |  |
| **Energy intake** |  |  |  |  |  | 0.015 |
| Yes | 8 | 0.93 (0.84, 1.02) | 83.1% | <0.001 | 0.116 |  |
| No | 2 | 0.71 (0.58, 0.86) | 0.0% | 0.452 | <0.001 |  |
| Abbreviations: N: number; CI, confidence interval; RR: relative risk  † P heterogeneity within subgroups.  * P for subgroup difference was calculated using Cochran’s Q test | | | | | | |

| **Supplementary Table 5.**Subgroup analysis of tea intake and depressive symptoms in cohort studies (highest versus lowest category meta-analysis, Relative risks and 95% confidence intervals) | | | | | | |
| --- | --- | --- | --- | --- | --- | --- |
| **Subgroup** | **n** | **RR**  **(95% CI)** | **I^2^**  **(%)** | ***P* Heterogeneity** | **P within**^†^ | ***P* Between*** |
| **Country** |  |  |  |  |  | 0.001 |
| European and American | 2 | 1.14 (0.99, 1.32) | 0.0% | 0.486 | 0.063 |  |
| Asian | 3 | 0.73 (0.58, 0.92) | 67.3% | 0.047 | 0.009 |  |
| **Follow-up duration** |  |  |  |  |  | 0.001 |
| <11 years | 3 | 0.73 (0.58, 0.92) | 67.3% | 0.047 | 0.009 |  |
| >11 years | 2 | 1.14 (0.99, 1.32) | 0.0% | 0.486 | 0.063 |  |
| **Number of participants** |  |  |  |  |  | 0.043 |
| <2000 | 3 | 0.84 (0.67, 1.04) | 71.6% | 0.030 | 0.116 |  |
| >2000 | 2 | 1.10 (0.95, 1.27) | 82.7% | 0.016 | 0.197 |  |
| **Alcohol consumption** |  |  |  |  |  | 0.008 |
| Yes | 3 | 1.12 (0.97, 1.28) | 68.8% | 0.041 | 0.127 |  |
| No | 2 | 0.77 (0.60, 0.98) | 71.9% | 0.059 | 0.030 |  |
| **BMI** |  |  |  |  |  | 0.001 |
| Yes | 2 | 1.14 (0.99, 1.32) | 0.0% | 0.486 | 0.063 |  |
| No | 3 | 0.73 (0.58, 0.92) | 67.3% | 0.047 | 0.009 |  |
| **Physical activity** |  |  |  |  |  | 0.248 |
| Yes | 3 | 1.06 (0.92, 1.22) | 84.6% | 0.002 | 0.428 |  |
| No | 2 | 0.90 (0.71, 1.14) | 61.3% | 0.108 | 0.384 |  |
| **Smoking** |  |  |  |  |  | 0.008 |
| Yes | 3 | 1.12 (0.97, 1.28) | 68.8% | 0.041 | 0.127 |  |
| No | 2 | 0.77 (0.60, 0.98) | 71.9% | 0.059 | 0.030 |  |
| Abbreviations: N: number; CI, confidence interval; RR: relative risk  † P heterogeneity within subgroups.  * P for subgroup difference was calculated using Cochran’s Q test | | | | | | |


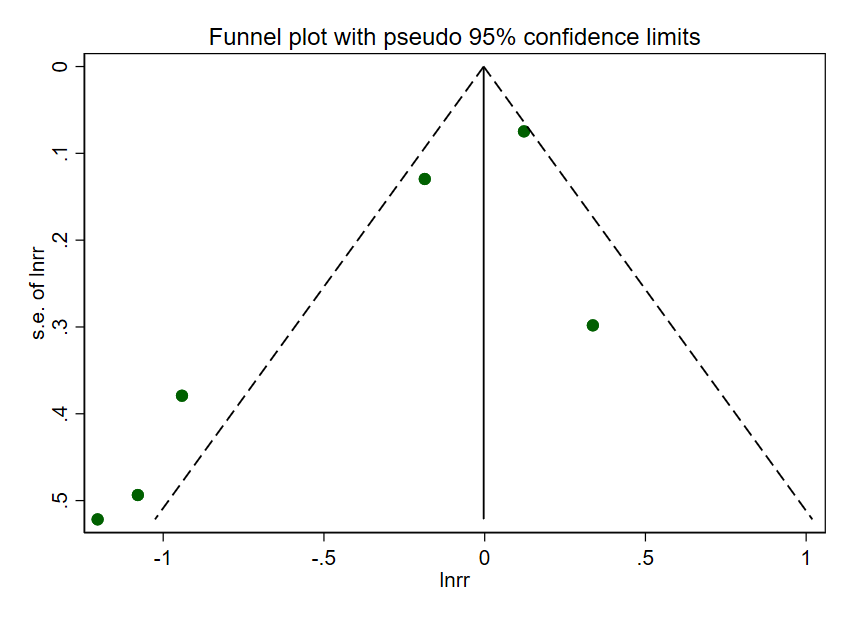


**Supplementary Figure 4.** Funnel plot of the relative risks of 6 cohort studies about tea intake (highest versus lowest category analysis) and risk of depressive symptoms. Egger test *P*=0.07. Log RR: natural logarithm of relative risk. s.e: standard error.


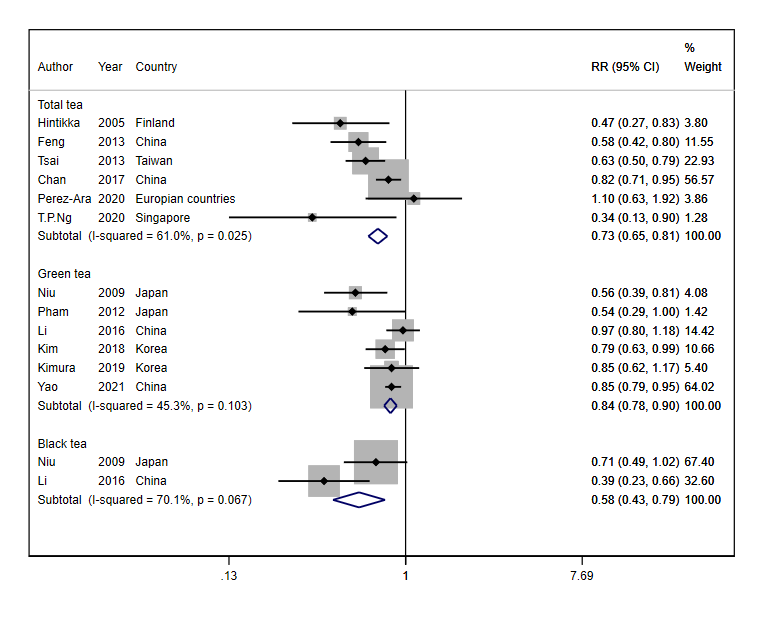


**Supplementary Figure 5.** Relative risk of depressive symptoms for the highest versus lowest category of total tea, green tea and black tea intake. RR, relative risk.

| **Supplementary Table 6.** Subgroup analysis of total tea and green tea intake and depressive symptoms in cross-sectional studies (highest versus lowest category meta-analysis, Relative risks and 95% confidence intervals) | | | | | | |
| --- | --- | --- | --- | --- | --- | --- |
| **Subgroup** | **n** | **RR**  **(95% CI)** | **I^2^**  **(%)** | ***P* Heterogeneity** | **P within**^†^ | ***P* Between**^*^ |
| **Total tea** |  | | | | | |
| **Number of participants** |  |  |  |  |  | 0.019 |
| <2000 | 3 | 0.79 (0.69, 0.90) | 61.1% | 0.077 | 0.001 |  |
| >2000 | 3 | 0.59 (0.48, 0.72) | 8.8% | 0.334 | <0.001 |  |
| **Alcohol consumption** |  |  |  |  |  | 0.521 |
| Yes | 3 | 0.65 (0.45, 0.93) | 68.9% | 0.040 | 0.020 |  |
| No | 3 | 0.73 (0.65, 0.82) | 66.6% | 0.050 | <0.001 |  |
| **BMI** |  |  |  |  |  | 0.973 |
| Yes | 2 | 0.72 (0.49, 1.07) | 77.5% | 0.035 | 0.106 |  |
| No | 4 | 0.73 (0.65, 0.81) | 64.2% | 0.039 | <0.001 |  |
| **Physical activity** |  |  |  |  |  | 0.109 |
| Yes | 4 | 0.61 (0.48, 0.78) | 54.7% | 0.085 | <0.001 |  |
| No | 2 | 0.76 (0.67, 0.86) | 72.5% | 0.057 | <0.001 |  |
| **Smoking** |  |  |  |  |  | 0.521 |
| Yes | 3 | 0.65 (0.45, 0.93) | 68.9% | 0.040 | 0.020 |  |
| No | 3 | 0.73 (0.65, 0.82) | 66.6% | 0.050 | 0.001 |  |
| **Green tea** |  | | | | | |
| **Number of participants** |  |  |  |  |  | 0.008 |
| <1500 | 2 | 0.55 (0.40, 0.76) | 0.0% | 0.921 | 0.001 |  |
| >1500 | 4 | 0.86 (0.80, 0.93) | 0.0% | 0.555 | <0.001 |  |
| **Energy intake** |  |  |  |  |  | 0.085 |
| Yes | 2 | 0.72 (0.59, 0.87) | 59.4% | 0.117 | 0.001 |  |
| No | 4 | 0.86 (0.80, 0.93) | 19.1% | 0.295 | <0.001 |  |
| Abbreviations: N: number; CI, confidence interval; RR: relative risk  † P heterogeneity within subgroups.  * P for subgroup difference was calculated using Cochran’s Q test | | | | | | |


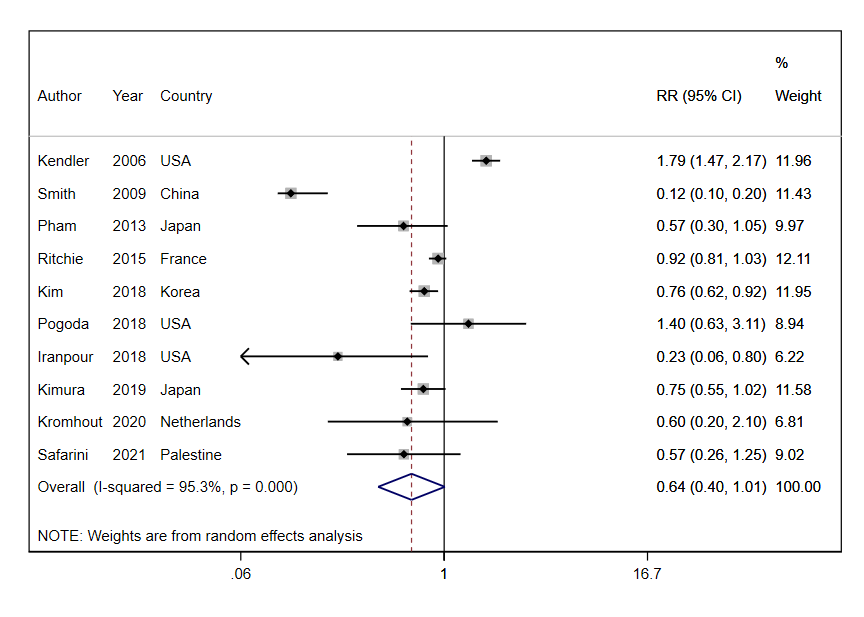
 **Supplementary Figure 6.** Relative risk of depressive symptoms for the highest versus lowest category of caffeine intake. RR, relative risk.


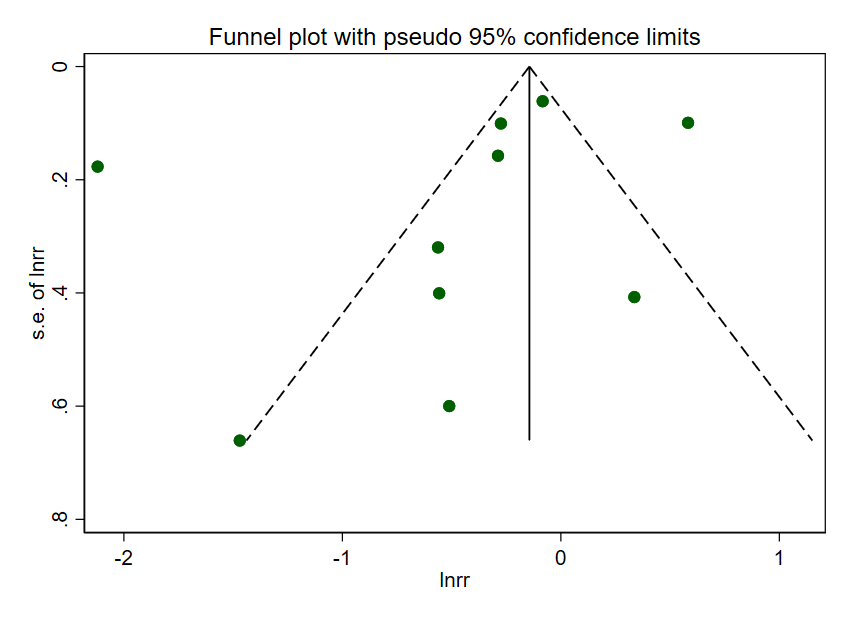
**Supplementary Figure 7.** Funnel plot of the relative risks of 10 cross-sectional studies about caffeine intake (highest versus lowest category analysis) and risk of depressive symptoms. Egger test *P*=0.34. Log RR: natural logarithm of relative risk. s.e: standard error.

**Supplementary Table 7.** GRADE evidence table for association of dietary caffeine, coffee, tea consumption and depressive symptoms in adults.

| **Certainty assessment** | | | | | | | **№ of patients** | | **Effect** | | **Certainty** | **Importance** |
| --- | --- | --- | --- | --- | --- | --- | --- | --- | --- | --- | --- | --- |
| **№ of studies** | **Study design** | **Risk of bias** | **Inconsistency** | **Indirectness** | **Imprecision** | **Other considerations** | **Participants** | **Case** | **Relative (95% CI)** | **Absolute (95% CI)** |  |  |
| **Coffee** | | | | | | | | | | | | |
| 4 | observational studies | serious^a^ | serious^b^ | not serious | not serious | publication bias strongly suspected dose response gradient^c^ | 319996 | 13583/319996 (4.2%) | **RR 0.89** (0.82 to 0.95) | **5 fewer per 1,000** (from 8 fewer to 2 fewer) | ⨁⨁◯◯ Low | IMPORTANT |
| **Tea** | | | | | | | | | | | | |
| 6 | observational studies | very serious^d^ | serious^e^ | not serious | very serious^f^ | none | 263180 | 12471/263180 (4.7%) | **RR 0.74** (0.51 to 1.08) | **12 fewer per 1,000** (from 23 fewer to 4 more) | ⨁◯◯◯ Very low | IMPORTANT |
| **Dietary caffeine** | | | | | | | | | | | | |
| 2 | observational studies | very serious^d^ | not serious | not serious | not serious | dose response gradient | 8017 | 1125/8017 (14.0%) | **RR 0.86** (0.79 to 0.93) | **20 fewer per 1,000** (from 29 fewer to 10 fewer) | ⨁⨁⨁◯ Moderate | IMPORTANT |

**CI:** confidence interval; **RR:** risk ratio

**Explanations**

a. Serious risk of bias since half of studies were at high risk of bias. Downgraded.

b. Serious inconsistency since I2=63.7%, Phet=0.04. Downgraded.

c. Visual inspection of funnel plot and Egger’s regression test (P<0.001) revealed a significant publication bias among included studies. Downgraded.

d. Very serious risk of bias since most of studies were at high risk of bias. Downgraded.

e. Serious inconsistency since I2=77.6%, Phet=0.001. Downgraded.

f. Very serious imprecision since the 95%CI include the null value and bounds of the 95%CI <0.9 and 1.1, and wide confidence interval. Downgraded


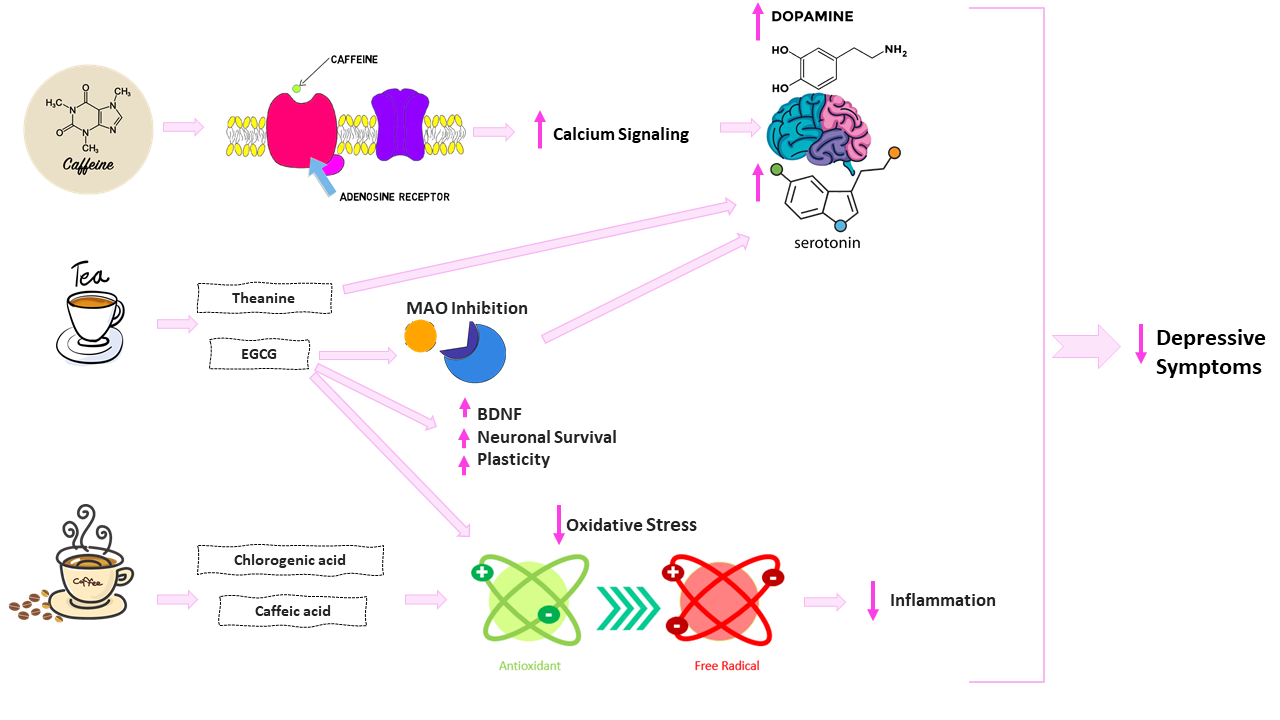
**Supplementary Figure 8.** Mechanisms of the effects of the dietary compounds on brain functions.

| **Section and Topic** | **Item #** | **Checklist item** | **Location where item is reported** |
| --- | --- | --- | --- |
| **TITLE** | | |  |
| Title | 1 | Identify the report as a systematic review. | 1-2 |
| **ABSTRACT** | | |  |
| Abstract | 2 | See the PRISMA 2020 for Abstracts checklist. | 2-3 |
| **INTRODUCTION** | | |  |
| Rationale | 3 | Describe the rationale for the review in the context of existing knowledge. | 4-5 |
| Objectives | 4 | Provide an explicit statement of the objective(s) or question(s) the review addresses. | 5 |
| **METHODS** | | |  |
| Eligibility criteria | 5 | Specify the inclusion and exclusion criteria for the review and how studies were grouped for the syntheses. | 5-6 |
| Information sources | 6 | Specify all databases, registers, websites, organisations, reference lists and other sources searched or consulted to identify studies. Specify the date when each source was last searched or consulted. | 5 |
| Search strategy | 7 | Present the full search strategies for all databases, registers and websites, including any filters and limits used. | 5 |
| Selection process | 8 | Specify the methods used to decide whether a study met the inclusion criteria of the review, including how many reviewers screened each record and each report retrieved, whether they worked independently, and if applicable, details of automation tools used in the process. | 6 |
| Data collection process | 9 | Specify the methods used to collect data from reports, including how many reviewers collected data from each report, whether they worked independently, any processes for obtaining or confirming data from study investigators, and if applicable, details of automation tools used in the process. | 6 |
| Data items | 10a | List and define all outcomes for which data were sought. Specify whether all results that were compatible with each outcome domain in each study were sought (e.g. for all measures, time points, analyses), and if not, the methods used to decide which results to collect. | 6 |
|  | 10b | List and define all other variables for which data were sought (e.g. participant and intervention characteristics, funding sources). Describe any assumptions made about any missing or unclear information. | 6 |
| Study risk of bias assessment | 11 | Specify the methods used to assess risk of bias in the included studies, including details of the tool(s) used, how many reviewers assessed each study and whether they worked independently, and if applicable, details of automation tools used in the process. | 6-7 |
| Effect measures | 12 | Specify for each outcome the effect measure(s) (e.g. risk ratio, mean difference) used in the synthesis or presentation of results. | 6-7 |
| Synthesis methods | 13a | Describe the processes used to decide which studies were eligible for each synthesis (e.g. tabulating the study intervention characteristics and comparing against the planned groups for each synthesis (item #5)). | 7-8 |
|  | 13b | Describe any methods required to prepare the data for presentation or synthesis, such as handling of missing summary statistics, or data conversions. | 7-8 |
|  | 13c | Describe any methods used to tabulate or visually display results of individual studies and syntheses. | 7-8 |
|  | 13d | Describe any methods used to synthesize results and provide a rationale for the choice(s). If meta-analysis was performed, describe the model(s), method(s) to identify the presence and extent of statistical heterogeneity, and software package(s) used. | 7-8 |
|  | 13e | Describe any methods used to explore possible causes of heterogeneity among study results (e.g. subgroup analysis, meta-regression). | 7-8 |
|  | 13f | Describe any sensitivity analyses conducted to assess robustness of the synthesized results. | 7-8 |
| Reporting bias assessment | 14 | Describe any methods used to assess risk of bias due to missing results in a synthesis (arising from reporting biases). | 7-8 |
| Certainty assessment | 15 | Describe any methods used to assess certainty (or confidence) in the body of evidence for an outcome. | 8 |
| **RESULTS** | | |  |
| Study selection | 16a | Describe the results of the search and selection process, from the number of records identified in the search to the number of studies included in the review, ideally using a flow diagram. | 9 |
|  | 16b | Cite studies that might appear to meet the inclusion criteria, but which were excluded, and explain why they were excluded. | 9 |
| Study characteristics | 17 | Cite each included study and present its characteristics. | 9-10 |
| Risk of bias in studies | 18 | Present assessments of risk of bias for each included study. | 10 |
| Results of individual studies | 19 | For all outcomes, present, for each study: (a) summary statistics for each group (where appropriate) and (b) an effect estimate and its precision (e.g. confidence/credible interval), ideally using structured tables or plots. | Table 1 |
| Results of syntheses | 20a | For each synthesis, briefly summarise the characteristics and risk of bias among contributing studies. | 11-14 |
|  | 20b | Present results of all statistical syntheses conducted. If meta-analysis was done, present for each the summary estimate and its precision (e.g. confidence/credible interval) and measures of statistical heterogeneity. If comparing groups, describe the direction of the effect. | 11-14 |
|  | 20c | Present results of all investigations of possible causes of heterogeneity among study results. | 11-14 |
|  | 20d | Present results of all sensitivity analyses conducted to assess the robustness of the synthesized results. | 11-14 |
| Reporting biases | 21 | Present assessments of risk of bias due to missing results (arising from reporting biases) for each synthesis assessed. | 11-14 |
| Certainty of evidence | 22 | Present assessments of certainty (or confidence) in the body of evidence for each outcome assessed. | 14  Table S5 |
| **DISCUSSION** | | |  |
| Discussion | 23a | Provide a general interpretation of the results in the context of other evidence. | 14-15 |
|  | 23b | Discuss any limitations of the evidence included in the review. | 16-17 |
|  | 23c | Discuss any limitations of the review processes used. | 16-17 |
|  | 23d | Discuss implications of the results for practice, policy, and future research. | 17 |
| **OTHER INFORMATION** | | |  |
| Registration and protocol | 24a | Provide registration information for the review, including register name and registration number, or state that the review was not registered. | 5 |
|  | 24b | Indicate where the review protocol can be accessed, or state that a protocol was not prepared. | 5 |
|  | 24c | Describe and explain any amendments to information provided at registration or in the protocol. | 5 |
| Support | 25 | Describe sources of financial or non-financial support for the review, and the role of the funders or sponsors in the review. | 17 |
| Competing interests | 26 | Declare any competing interests of review authors. | 18 |
| Availability of data, code and other materials | 27 | Report which of the following are publicly available and where they can be found: template data collection forms; data extracted from included studies; data used for all analyses; analytic code; any other materials used in the review. | 18 |

*From:*  Page MJ, McKenzie JE, Bossuyt PM, Boutron I, Hoffmann TC, Mulrow CD, et al. The PRISMA 2020 statement: an updated guideline for reporting systematic reviews. BMJ 2021;372:n71. doi: 10.1136/bmj.n71

For more information, visit: <http://www.prisma-statement.org/>

| **Section and Topic** | **Item #** | **Checklist item** | **Reported (Yes/No)** |
| --- | --- | --- | --- |
| **TITLE** | | |  |
| Title | 1 | Identify the report as a systematic review. | Yes |
| **BACKGROUND** | | |  |
| Objectives | 2 | Provide an explicit statement of the main objective(s) or question(s) the review addresses. | Yes |
| **METHODS** | | |  |
| Eligibility criteria | 3 | Specify the inclusion and exclusion criteria for the review. | Yes |
| Information sources | 4 | Specify the information sources (e.g. databases, registers) used to identify studies and the date when each was last searched. | Yes |
| Risk of bias | 5 | Specify the methods used to assess risk of bias in the included studies. | Yes |
| Synthesis of results | 6 | Specify the methods used to present and synthesise results. | Yes |
| **RESULTS** | | |  |
| Included studies | 7 | Give the total number of included studies and participants and summarise relevant characteristics of studies. | Yes |
| Synthesis of results | 8 | Present results for main outcomes, preferably indicating the number of included studies and participants for each. If meta-analysis was done, report the summary estimate and confidence/credible interval. If comparing groups, indicate the direction of the effect (i.e. which group is favoured). | Yes |
| **DISCUSSION** | | |  |
| Limitations of evidence | 9 | Provide a brief summary of the limitations of the evidence included in the review (e.g. study risk of bias, inconsistency and imprecision). | Yes |
| Interpretation | 10 | Provide a general interpretation of the results and important implications. | Yes |
| **OTHER** | | |  |
| Funding | 11 | Specify the primary source of funding for the review. | Yes |
| Registration | 12 | Provide the register name and registration number. | Yes |

*From:*  Page MJ, McKenzie JE, Bossuyt PM, Boutron I, Hoffmann TC, Mulrow CD, et al. The PRISMA 2020 statement: an updated guideline for reporting systematic reviews. BMJ 2021;372:n71. doi: 10.1136/bmj.n71

For more information, visit: <http://www.prisma-statement.org/>
